# Consolidated data on the phylogeny and evolution of the family Tritoniidae (Gastropoda: Nudibranchia) contribute to genera reassessment and clarify the taxonomic status of the neuroscience models *Tritonia* and *Tochuina*

**DOI:** 10.1371/journal.pone.0242103

**Published:** 2020-11-20

**Authors:** Tatiana Korshunova, Alexander Martynov

**Affiliations:** 1 Koltzov Institute of Developmental Biology, Moscow, Russia; 2 Zoological Museum of the Moscow State University, Moscow, Russia; Laboratoire de Biologie du Développement de Villefranche-sur-Mer, FRANCE

## Abstract

Nudibranch molluscs of the family Tritoniidae are widely used neuroscience model systems for understand the behavioural and genetic bases of learning and memory. However species identity and genus-level taxonomic assignment of the tritoniids remain contested. Herein we present a taxonomic review of the family Tritoniidae using integration of molecular phylogenetic analysis, morphological and biogeographical data. For the first time the identity of the model species *Tritonia tetraquetra* (Pallas, 1788) and *Tritonia exsulans* Bergh, 1894 is confirmed. *T*. *tetraquetra* distributes across the large geographic and bathymetric distances in the North-Eastern (NE) and North-Western (NW) Pacific. In turn, at NE Pacific coasts the separate species *T*. *exsulans* is commonly occured. Thus, it reveals a misidentification of *T*. *tetraquetra* and *T*. *exsulans* species in neuroscience applications. Presence of more hidden lineages within NW Pacific *T*. *tetraquetra* is suggested. The long lasting confusion over identity of the species from the genera *Tritonia* and *Tochuina* is resolved using molecular and morphological data. We also disprove a common indication about “edible *T*. *tetraquetra*” at the Kuril Islands. It is shown that *Tochuina* possesses specialized tritoniid features and also some characters of “arminacean nudibranchs”, such as *Doridoxa* and *Heterodoris*. Diagnoses for the families Doridoxidae and Heterodorididae are provided. Taxonomy of the genus *Doridoxa* is clarified and molecular data for the genus *Heterodoris* presented for the first time. A taxonomic synopsis for the family Tritoniidae is provided. A new genus among tritoniid taxa is proposed. Importance of the ontogeny-based taxonomy is highlighted. The cases when apomorphic characters considerably modified in a crown group due to the paedomorphosis are revealed. Tracing of the character evolution is presented for secondary gills–a key external feature of the family Tritoniidae and traditional dendronotacean nudibranchs.

## Introduction

A remarkable nudibranch family Tritoniidae has an intricate phylogenetic position within Nudibranchia [1–5]. For several decades species of the genera *Tritonia* and *Tochuina* have been no less useful model systems for studies neural basis of behaviour than *Aplysia* [6–12]. The brain of tritoniids contains giant neurons which can be reliably identified by their behavioural functions. Studies of how the brain controls behaviour are conducting, as well through identify homologous neurons in different mollusc taxa [10–13]. It can shed light on evolution the neural basis of behaviour but requires precise taxa identification.

**Fig 1:**
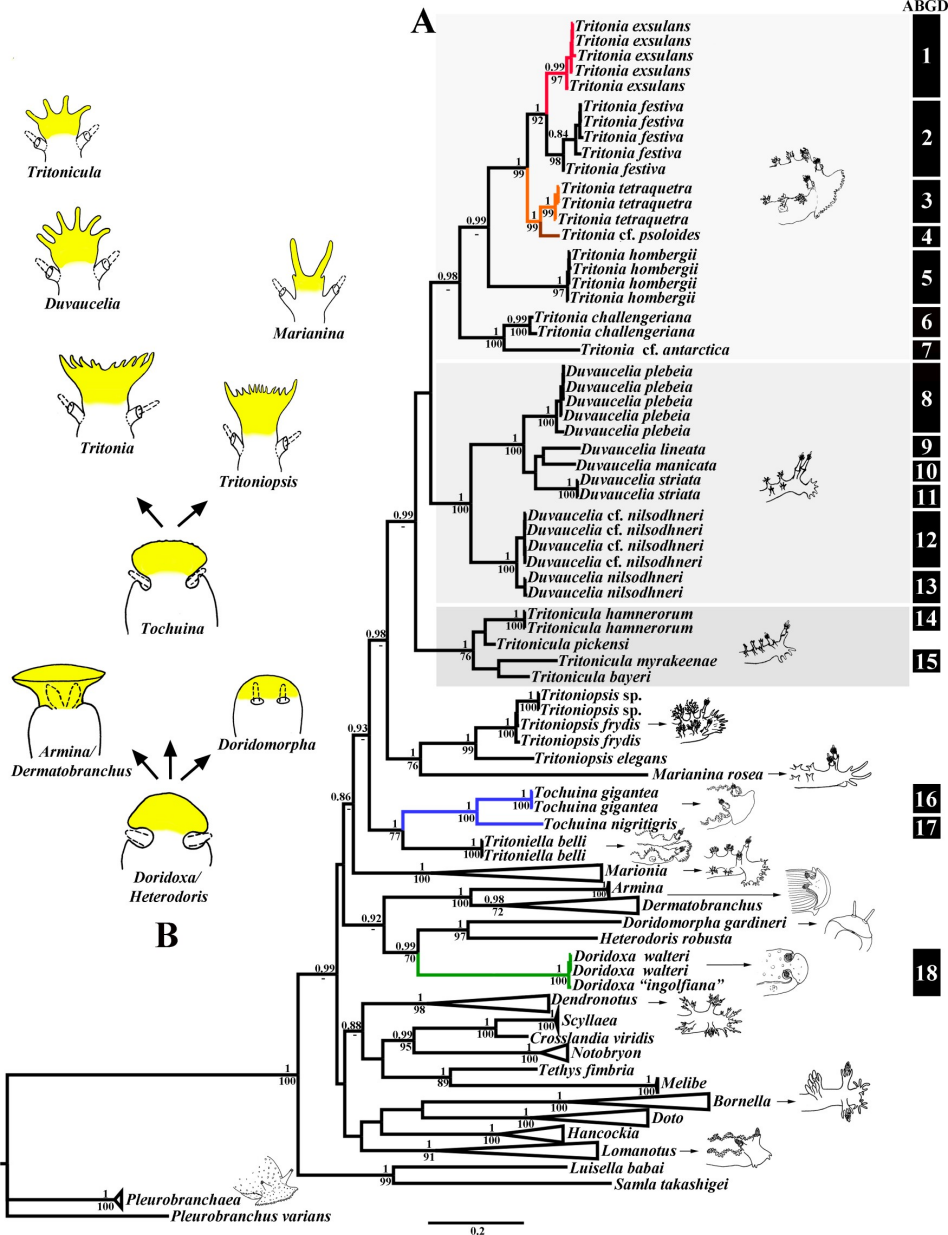
A. Phylogenetic relationships of Tritoniidae, Doridoxidae, Heterodorididae, Doridomorphidae, Arminidae and Dendronotoidea based on COI + 16S + H3 concatenated dataset inferred by Bayesian inference (BI). Numbers above branches represent posterior probabilities from BI; numbers below branches indicate bootstrap values for Maximum Likelihood. B. Scheme of the potential morphological transformations of the rhinophores and oral veil that supported by the present phylogenetic data (see discussion in the text). Oral veil and derivates highlighted in yellow colour.

**Fig 2:**
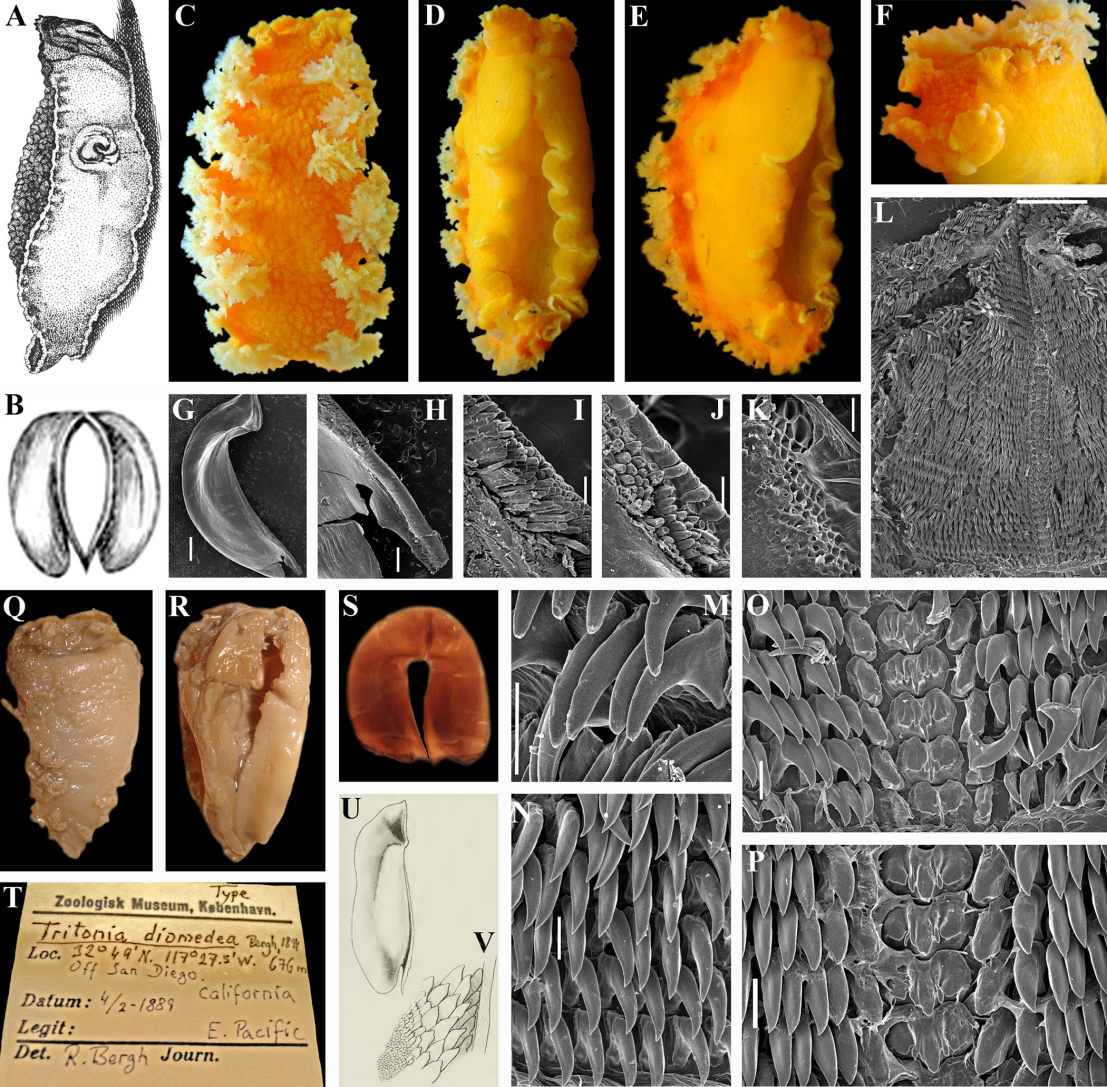
*Tritonia tetraquetra* (Pallas, 1788). A. Figure of high body (laterodorsal view) with bilobed oral veil and without distinct notal edge of “*Limax*” *tetraquetra* from the original description by Pallas (1788 [38]). B. Figure of elongate-oval jaws of “*Limax*” *tetraquetra* from the original description by Pallas (1788 [38]). C–E. Dorsal, ventral and lateral views respectively of living neotype (ZMMU Op-719) of *Tritonia tetraquetra* from Kamchatka (120 mm in length). F. Details of bilobed oral veil of living neotype of *T*. *tetraquetra*. G. Elongate-oval jaw of specimen *T*. *tetraquetra* from Kamchatka (length 170 mm, live), collected on the same date with neotype (SEM). H. Same specimen, overview of masticatory process of jaw. I, J. Same specimen, details of elongate britsle-like elements of masticatory process. K. Same, details of polygonal structures on the masticatory process. L. Same specimen, general overview of radula (SEM). M. Same, details of outer lateral teeth. N. Same, details of middle lateral teeth. O, P. Same, details of central part of the radula with central teeth and inner laterals. Q, R. Dorsal and and ventral views respectively of the lectotype *T*. *diomedea* Bergh, 1894 (junior synonym of *T*. *tetraquetra*) (ZMUK, GAS-2034, 30 mm in length). S. Elongate-oval jaws of lectotype *T*. *diomedea*. T. Label of the type of *T*. *diomedea* (ZMUK, GAS-2034). U. Jaw from first description of *T*. *diomedea* (from Bergh, 1894, [50]). V. Details of details of elongate britsle-like elements of masticatory process from the first description of *T*. *diomedea*. Scale bars: G, L– 2 mm, H– 500 μm, I–K– 100 μm, M–P– 200 μm. All bibliographic excerpts are not in copyright.

**Fig 3:**
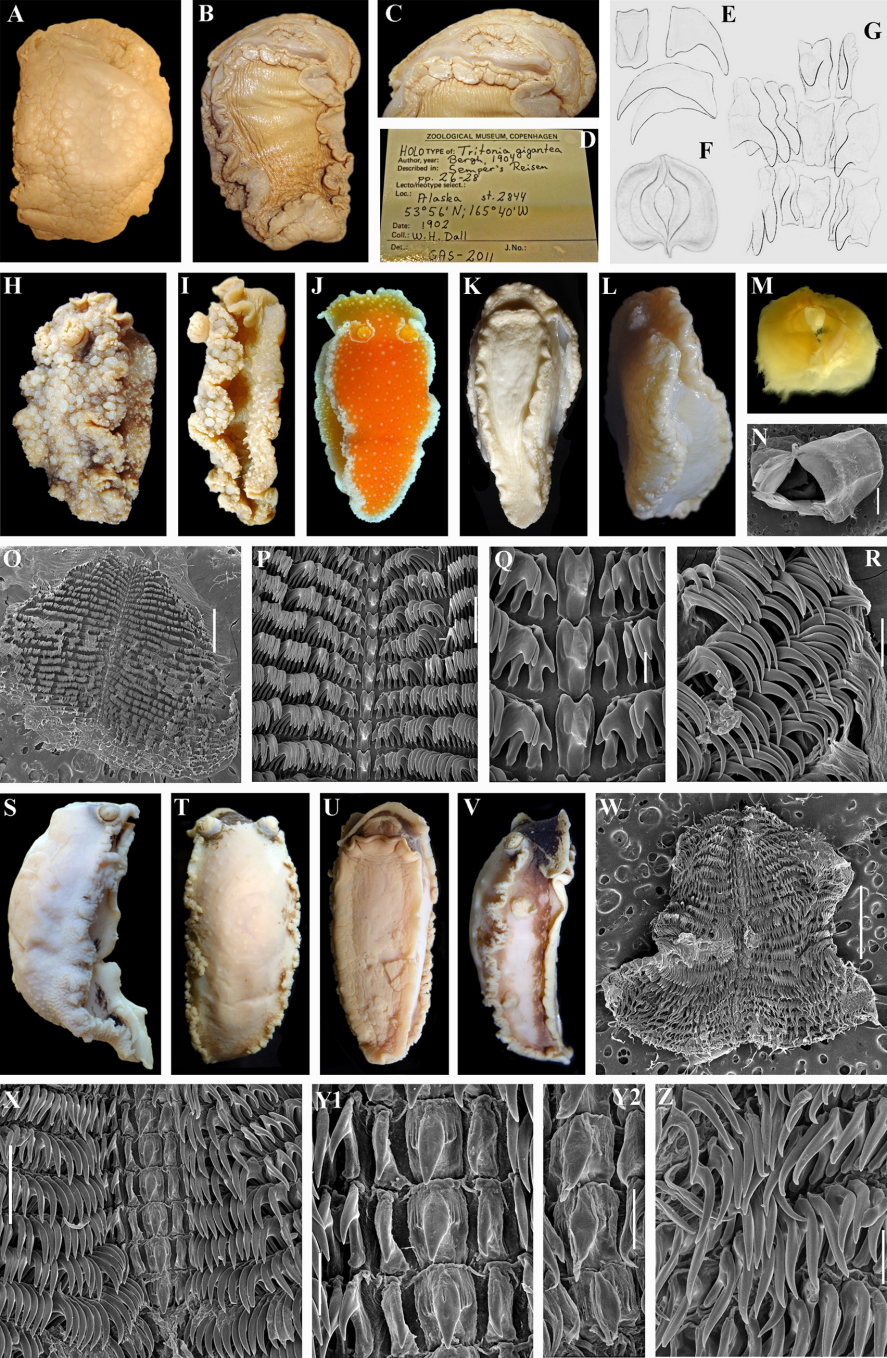
*Tochuina gigantea* (Bergh, 1904) *and Tochuina nigromaculata* (Roginskaya, 1984) comb. nov. A, B. *Tochuina gigantea*, dorsal and ventral views respectively of the preserved holotype (ZMUC GAS-2011, length 120 mm). C. Oral veil (not bilobed, ventral view) of the preserved holotype of *T*. *gigantea*. D. Label of the holotype of *T*. *gigantea*. E. Central and lateral teeth from the first description *T*. *gigantea* in Bergh, 1904 [43]. F. Squarish jaws of *T*. *gigantea* from the work by Bergh (1879 [42], incorrect identification of *Tochuina gigantea* as “*Tritonia tetraquetra*”, see text for details). G. Central and inner lateral teeth of *T*. *gigantea* from the work by Bergh (1879 [42], incorrect identification of *Tochuina gigantea* as “*Tritonia tetraquetra*”). H, I. *T*. *gigantea*, dorsal and lateral views respectively of the preserved specimen from Okhotsk Sea (NW Pacific) (length 31 mm) showing low body with distinct notal edge. J–R. External views and internal characters of the specimen of *T*. *gigantea* from British Columbia (NE Pacific, 60 mm length, live, ZMMU Op-726). J. Dorsal view (live) showing partly opened rhinophoral sheath. K, L. Ventral and lateral views respectively (preserved). M. Squarish jaws (light microscopy). N. Jaws (SEM). O. General overview of radula (SEM). P. Central part of the radula with central and lateral teeth. Q. Details of the central teeth and inner laterals. R. Details of outer lateral teeth. S. *Tochuina nigromaculata*, lateral view of preserved specimen, 33 mm length, from Okhotsk Sea. T–Z. *T*. *nigromaculata*, external views and internal characters of paratype (ZMMU Op-746, 34 mm length, preserved) from the Pacific side of the Kuril Islands (off Simushir Island). T–V. Dorsal, ventral and lateral views respectively (preserved). W. General overview of radula (SEM). X. Central part of the radula with central and lateral teeth. Y1. Details of central teeth and inner laterals of the anterior and middle part of the radula. Y2. Details of central teeth and inner laterals of the posterior part of the radula. Z. Outer lateral teeth. Scale bars: N, O, W– 1 mm, P– 200 μm, Q– 50 μm, R, Y, Z– 100 μm, X– 300 μm. All bibliographic excerpts are not in copyright.

**Fig 4:**
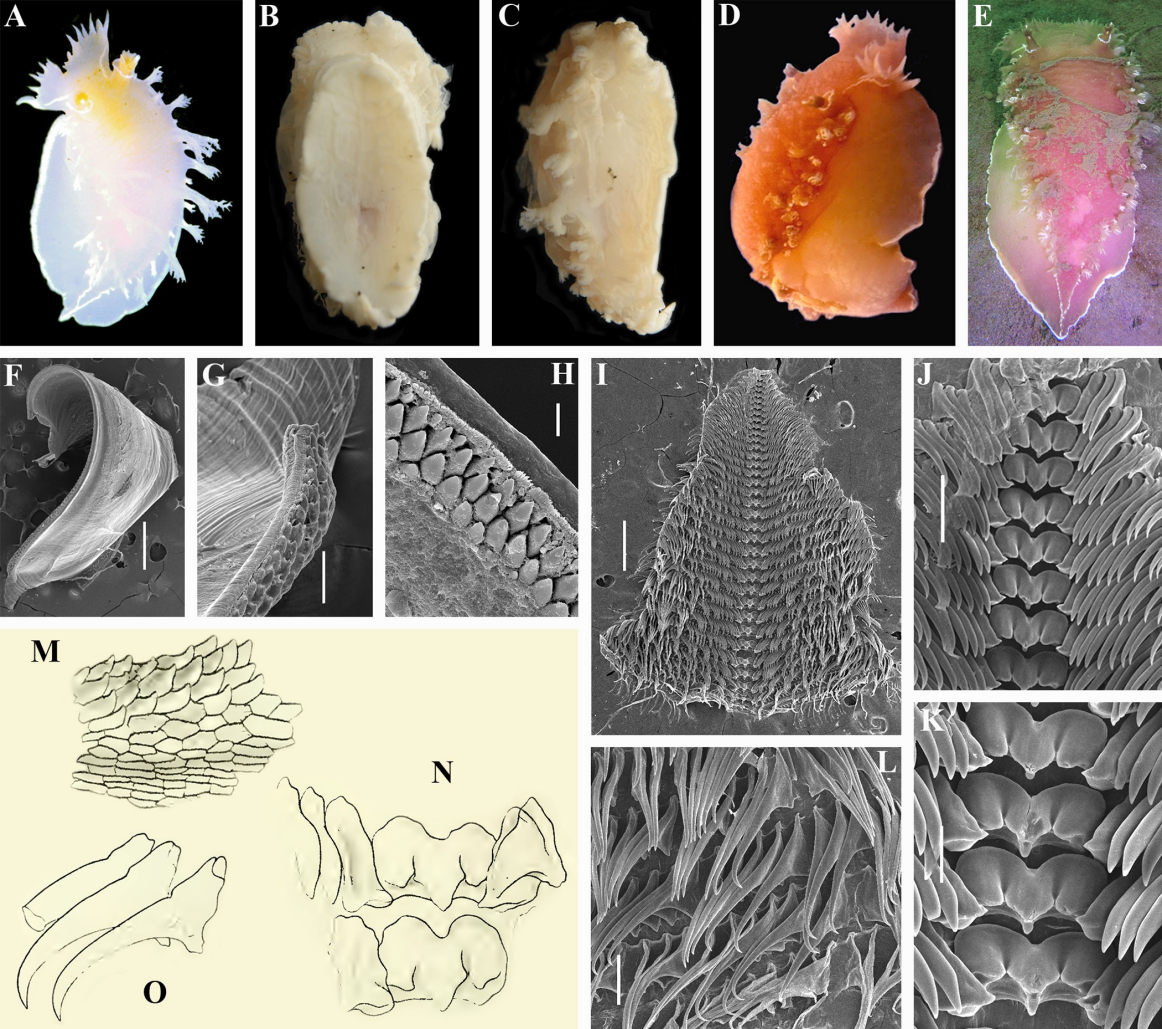
*Tritonia exsulans* Bergh, 1894. A. Dorsal view of living neotype of *T*. *exsulans* (NE Pacific) (ZMMU Op-720). B, C. Ventral and lateral views of preserved neotype of *T*. *exsulans* (13 mm in length). D, E. Living specimen of *T*. *exsulans* from NE Pacific before involvement to the neurobiological experiments. F. Jaw of neotype (SEM). G. Masticatory process of the same jaw. H. Details of massive conical elements of masticatory process of the same jaw. I. General overview of radula of neotype (SEM). J. Details of central part of the radula with central teeth and inner laterals. K. Details of central teeth. L. Details of outer laterals. M. Details of massive conical elements of masticatory process in the first description of *T*. *exsulans* (from Bergh, 1894 [50]). N. Central teeth in the first description of *T*. *exsulans* (from Bergh, 1894 [50]). O. Lateral teeth in the first description of *T*. *exsulans* (from Bergh, 1894 [50]). Scale bars: F, I– 500 μm, G, J, L– 100 μm, H– 10 μm, K– 50 μm. All bibliographic excerpts are not in copyright.

**Fig 5:**
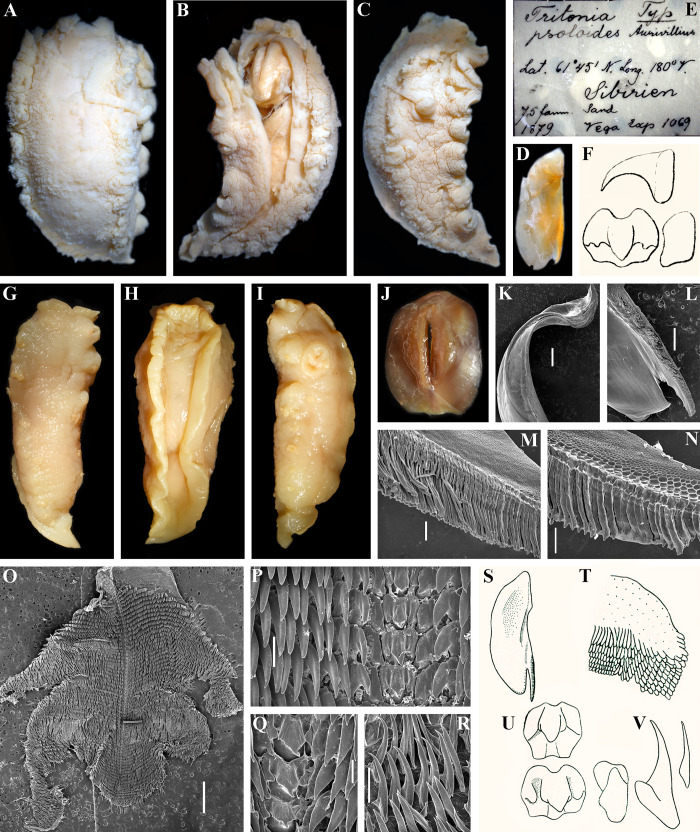
*Tritonia psoloides* Aurivillius, 1887 and *Tritonia primorjensis* Minichev, 1971. A–C. Dorsal, lateral and ventral views of the preserved type specimen of *T*. *psoloides* respectively (SMNH 6875, length ca. 45 mm). D. Jaw of type of *T*. *psoloides* (light microscopy). E. Label of the type of *T*. *psoloides* (SMNH 6875). F. Central and lateral teeth of *T*. *psoloides* from the original description in Aurivillius, 1887 [51]. G–R. External views and internal characters of the preserved specimen of *T*. *primorjensis* from the type locality in Peter the Great Bay (the Sea of Japan) which was collected in 70S–80S for neurobiological experiments (ZMMU Op-644, length 65 mm). G–I. Dorsal, ventral and lateral views respectively. J. Jaws (light microscopy). K. Part of jaw (SEM). L. Overview of masticatory process of jaw. M. Details of very elongate britsle-like elements of masticatory process. N. Details of jaws including polygonal structures on the masticatory process. O. General overview of radula (SEM). P. Details of central part of the radula with central teeth and inner laterals. Q. Details of central teeth. R. Outer lateral teeth. S. Jaw from the original descpription of *T*. *primorjensis* (from Minichev, 1971 [53]). T. Details of very elongate britsle-like elements of masticatory process from the original descripiton of *T*. *primorjensis*. U. Central and inner lateral teeth from the original descpription of *T*. *primorjensis*. V. Outer lateral teeth from the original descpription of *T*. *primorjensis*. Scale bars: K, O– 2 mm, L– 1mm, M, N, Q– 100 μm, P, R– 200 μm. All bibliographic excerpts are not in copyright.

**Fig 6:**
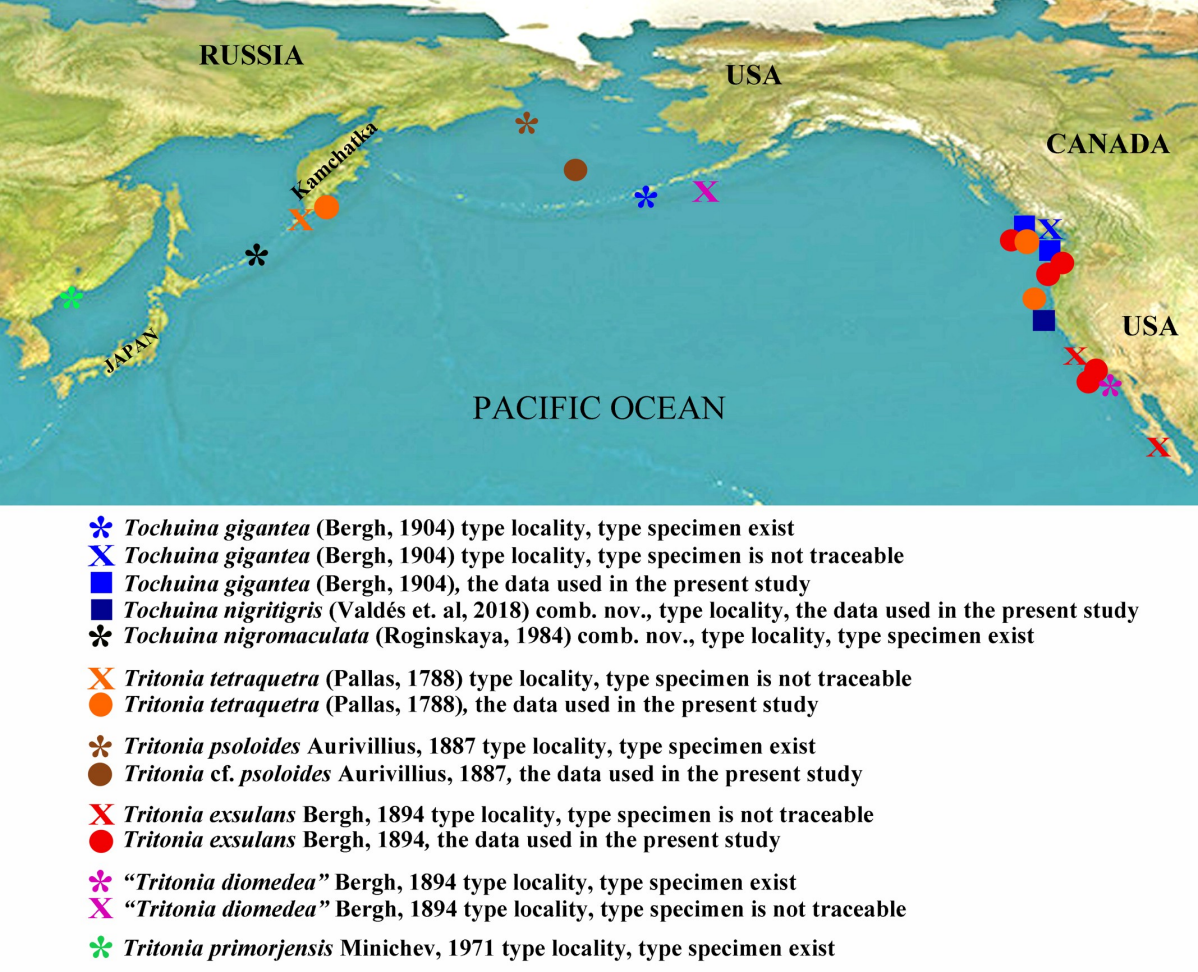
Distributional data for the taxa of North Pacific Tritoniidae involved in the present analysis with inclusion of the species of the genera *Tochuina* and *Tritonia* that were previously misidentified. See detailed explanations on the figure.

**Fig 7:**
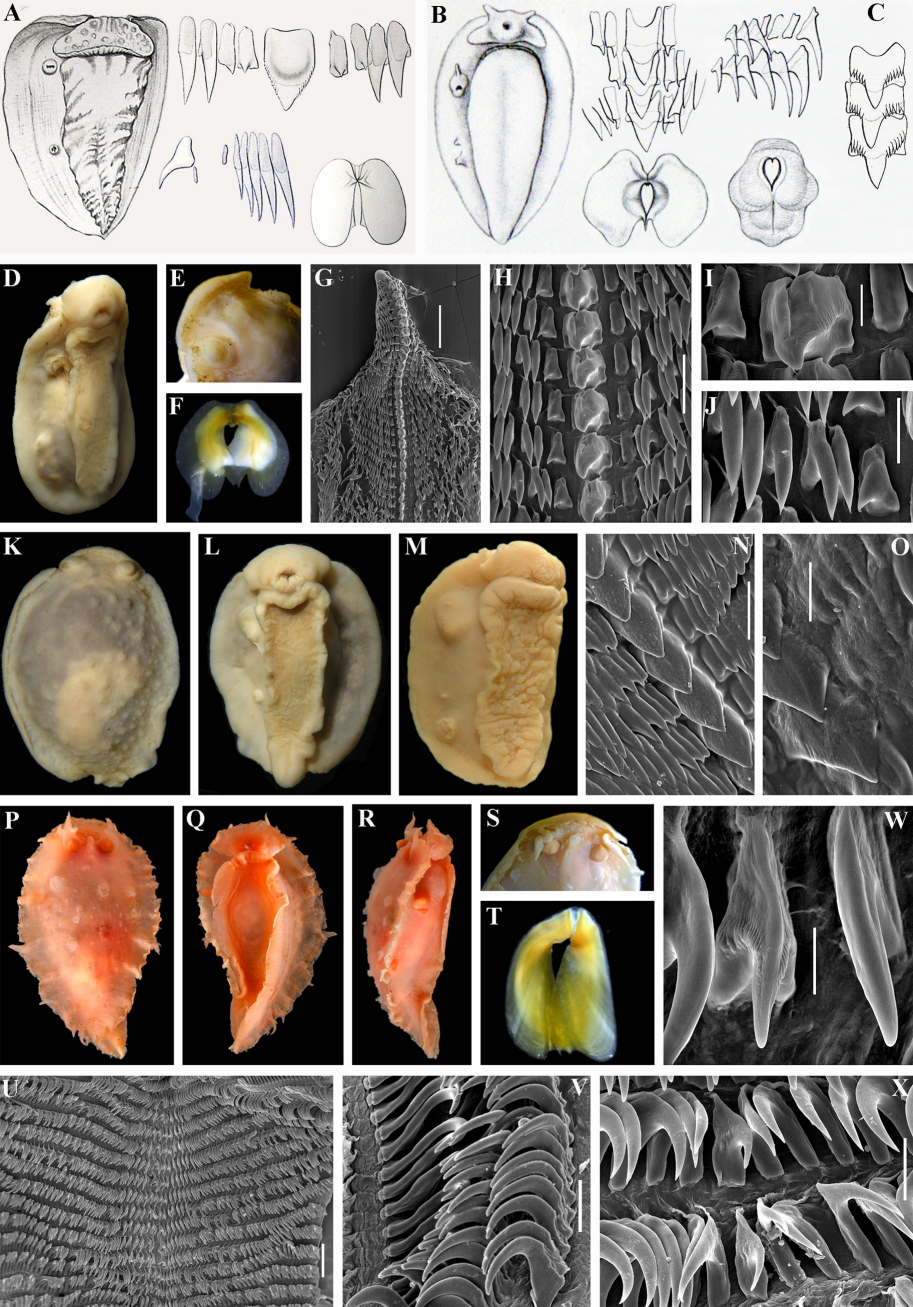
*Doridoxa walteri* (Krause, 1892) and *Heterodoris robusta* Verrill et Emerton 1882 –non-tritoniid taxa that showing external (presence of partly opened rhinophoral sheath connected to a non-bilobed oral veil) and internal characters (unicuspid central teeth, numerous hamate laterals) similar to the tritoniid genus *Tochuina*. A. *Doridoxa walteri*, morphological data from original description in Krause, 1892 [109], including external view, radular teeth and jaws. B. *Doridoxa ingolfiana* Bergh, 1899 (junior synonym of *D*. *walteri*, see text for details), morphological data from original description in Bergh, 1899 [105], including external view, radular teeth and jaws. C. “*Doridoxa ingolfiana* var.” from original description in Bergh, 1899 [105] (junior synonym of *D*. *walteri*, see text for details), showing denticulated central teeth. D–J. External views and internal characters of *Doridoxa walteri*, preserved specimen (ZMMU Op-721, length 16 mm) from the Barents Sea (eastern Spitzbergen region), type locality of *D*. *walteri*. D. Ventrolateral view. E. Details of oral veil connected to the partly opened rhinophoral sheath, essentially similar to the pattern of oral veil in *Tochuina*. F. Jaws (light microscopy). G. General overview of radula (SEM). H. Central part of the radula with central and lateral teeth showing presence of both denticulated and non denticulated central teeth. I. Details of the larger central teeth with distinct denticulation. J. Details of the smaller central teeth without denticulation. K, L. *D*. *walteri*, dorsal and ventral views respectively, preserved specimen from the Barents Sea (eastern Spitzbergen region, ZMMU Op-722, length 17 mm). M. *D*. *walteri*, lateroventral view, preserved specimen (length 14 mm) from the Barents Sea showing details of oral veil connected to the partly opened rhinophoral sheaths. N. Same specimen of *D*. *walteri*, central part of the radula with larger central teeth without distinct denticulation on lateral teeth. O. Same specimen of *D*. *walteri*, details of the larger central teeth with and without denticulation on the same teeth. P–X. External views and internal characters of *Heterodoris robusta*, specimen off Greenland and Canadian waters (ZMMU Op-723, length 39 mm) showing details of non bilobed oral veil connected to the partly opened rhinophoral sheaths. P–R. Dorsal, ventral and lateral views respectively (live). S. Details of oral veil (preserved). T. Jaws (light microscopy). U. General overview of radula with unicuspid central teeth and numerous hamate laterals, similar to the tritoniid *Tochuina* (SEM). V. Details of the outer lateral teeth. W. Details of the central part of the radula with central and inner lateral teeth. X. Central part of the radula with central and lateral teeth showing presence of both smooth and weakly denticulated central teeth. Scale bars: G, U– 300 μm, H, V, X– 100 μm, I, W– 30 μm, J– 50 μm, N– 80 μm, O– 40 μm. All bibliographic excerpts are not in copyright.

The most commonly used for neuroscience purposes was a North Pacific species previously known under the name *Tritonia diomedea* Bergh, 1894 [14–16], which recently was showed to be a junior synonym of *Tritonia tetraquetra* (Pallas, 1788) [17, 18]. However, though the latter name now is accepted [19–21] many questions have remained. Particularly, the large geographic and bathymetric ranges of *T*. *tetraquetra* in the northern Pacific were not tested using both morphological and molecular data. *Tritonia tetraquetra* is also frequently mentioned among few examples of apparent involvement of a nudibranch species in a culture of indigenous ethnic groups. This species was originally described from the Kuril Islands and the genus name *Tochuina* was proposed to be derived from Ainu language [22, 23]. Thus, members of the family Tritoniidae are important for several fields, however their taxonomic and phylogenetic placement needs in a clarification. Here we for the first time summarize available morphological, molecular and biogeographic data across the North-Eastern (NE) and the North-Western (NW) Pacific “*Tritonia tetraquetra”* group of species and the genus *Tochuina* to clarify their taxonomic status and phylogenetic relationships (Figs 1–7).

Recently we presented phylogenetic data and ancestral character state reconstruction for aeolidacean nudibranchs using the family Tritoniidae as an outgroup [24]. In order to place the model species of the genera *Tritonia* and *Tochuina* in a broad phylogenetic framework, in the present study we investigated the internal and external phylogenetic relations of the family Tritoniidae. This is particularly relevant because the tritoniid genus *Tochuina* shows an intriguing morphological similarity to the presumably distantly related non-tritoniid taxa *Doridoxa* and *Heterodoris* [25]. This similarity was never explored with application of the modern molecular data. Here we therefore present phylogenetic analysis with a broad taxon sampling and ancestral character state reconstruction for the superfamilies Dendronotoidea and Tritonioidea to trace the formation of key features of one of the most basal nudibranchs.

## Materials and methods

### Sample data

Material for this study was obtained by scuba diving in NW Pacific (Kamchatka, Russia, 52° 50´ N 158° 42´ E), NE Pacific (British Columbia, Canada, 50° 53´ N 125° 37´ W, 50° 36´ N, 126° 49´ W; Washington, USA, 47° 35´ N 122° 33´ W), NE Atlantic (Norway, 60° 57´ N 05° 07´ E), by dredging in NW Atlantic and the Barents Sea, and preserved in 99% ethanol for morphological and molecular investigations. For morphological study several previously collected formalin-fixed samples during dredging operations in the Sea of Japan (Peter the Great Bay) were used. Specimens are stored in the Zoological Museum of Moscow State University (ZMMU). Type specimens were examined from the Natural History Museum of Denmark and Swedish Museum of Natural History. No permission was necessary to obtain samples in the field and to access the museum collections.

### Morphological analysis

External and internal morphology was studied under a stereomicroscope, using a Nikon D-810 digital camera and scanning electron microscopes. The buccal masses were extracted and processed in 10% sodium hypochlorite solution to extract the radulae. Afterwards radulae and jaws were rinsed in the water and 70% ethanol, dried, mounted on stubs using carbon tape, coated with gold and palladium and examined using scanning electron microscopes (CamScan Series II, JSM 6380). The digital images were captured using a maximum quality mode (4) in CamScan II and 80-second capturing mode in JSM. The reproductive systems were examined using a stereomicroscope.

### Molecular analysis

For molecular analysis a small pieces were used for DNA extraction with Syntol S-Sorb kit by Syntol Company, according to the producer’s protocols. In total, 8 specimens were successfully sequenced for the mitochondrial genes cytochrome c oxidase subunit I (COI), 16S rRNA, and the nuclear genes Histone 3 (H3). Partial sequences were amplified by PCR using the primers: LCO 1490 (GGTCAACAAATCATAAAGATATTGG, [26]); HCO 2198 (TAAACTTCAGGGTGACCAAAAAATCA, [26]); 16 S arL (CGCCTGTTTAACAAAAACAT, [27]); 16 S R (CCGRTYTGAACTCAGCTCACG, [28]); H3 AF (ATGGCTCGTACCAAGCAGACGG, [29]) and H3 AR (ATATCCTTGGGCATGATGGTGAC, [29]). Extracted DNA was used as a template for the amplification of partial sequences. Polymerase chain reaction (PCR) amplifications were carried out in a 20 μL reaction volume, which included 4 μL of 5x Screen Mix (Eurogen Lab), 0.5 μL of each primer (10 μM stock), 1 μL of genomic DNA, and 14 μL of sterile water. The amplification of COI was performed with an initial denaturation for 1 min at 95° C, followed by 35 cycles of 15 secs at 95° C (denaturation), 15 secs at 45° C (annealing temperature), and 30 secs at 72° C, with a final extension of 7 mins at 72° C. The 16S amplification began with an initial denaturation for 1 min at 95° C, followed by 40 cycles of 15 secs at 95° C (denaturation), 15 secs at 52° C (annealing temperature), and 30 secs at 72° C, with a final extension of 7 mins at 72° C. The amplification of H3 began with an initial denaturation for 1 min at 95° C, followed by 40 cycles of 15 secs at 95° C (denaturation), 15 secs at 50° C (annealing temperature) and 30 secs at 72° C, with a final extension of 7 mins at 72° C. Sequencing for both strands proceeded with the ABI PRISM BigDye Terminator v.3.1. Sequencing reactions were analysed using an Applied Biosystems 3730 DNA Analyzer. Additional sequences were obtained from GenBank (see Table 1 for a full list of samples, localities, and voucher references). Original data and publicly available sequences were aligned with the MAFFT algorithm [30]. COI and H3 sequences were translated into amino acids for confirmation of the alignment. Separate analyses were conducted for COI (657 bp), 16S (425 bp), H3 (327 bp), and concatenated data (1409 bp). Gblocks 0.91b [31] was applied to discard poorly aligned regions for the 16S data set using less stringent options (allow smaller final blocks, gap positions within the final blocks, and less strict flanking positions), in total, 13% of the positions were eliminated. Evolutionary models for each data set were selected using MrModelTest 2.3 [32]. Two different phylogenetic methods, Bayesian inference (BI) and Maximum likelihood (ML) were used to infer evolutionary relationships. Bayesian estimation of posterior probability was performed in MrBayes 3.2 [33]. Four Markov chains were sampled at intervals of 1000 generations. Analysis was started with random starting trees and 5 × 10^6^ generations. Maximum likelihood-based phylogeny inference was performed in RAxML 7.2.8 [34] with bootstrap in 1000 pseudo-replications. Final phylogenetic tree images were rendered in FigTree1.4.2. Nodes in the phylogenetic trees with Bayesian posterior values ≥0.96 and bootstrap values ≥90 were considered ‘highly’ supported. Nodes with 0.90–0.95 and 80–89 accordingly were considered ‘moderately’ supported (lower support values were considered not significant). The program MEGA7 [35] was used to calculate the uncorrected p-distances between all the sequences. Automatic Barcode Gap Discovery (ABGD) [36] (https://bioinfo.mnhn.fr/abi/public/abgd/abgdweb.html) was used to estimate *Tritonia* sensu lato, *Tochuina* and *Doridoxa* species divergence. Alignment from the COI marker was submitted and processed in ABGD using the Jukes-Cantor (JC69) and Kimura (K80) models with the following settings: a prior for the maximum value of intraspecific divergence between 0.001 and 0.1, 10 recursive steps within the primary partitions defined by the first estimated gap, and a gap width of 1. Ancestral character state reconstruction for the secondary gill traits were run using Parsimony ancestral states in Mesquite v3.10 [37], based on the topology of the best tree from the Bayesian analysis of a concatenated dataset.

**Table 1:** GenBank accession numbers for all sequences used in this study.

Species name	Voucher	Locality	COI	16S	H3
*Armina scotti* Mehrotra, Caballer & Chavanich, 2017	-	Thailand	KX538792	KX538793	-
*Armina scotti* Mehrotra, Caballer & Chavanich, 2017	CASIZ177535	Philippines	HQ010504	HQ010539	HQ010473
*Armina scotti* Mehrotra, Caballer & Chavanich, 2017	CASIZ177534	Philippines	HM162696	HM162606	HM162512
*Bornella johnsonorum* Pola, Rudman & Gosliner, 2009	CASIZ175407	Marshall Islands	JN869445	JN869401	JN869419
*Bornella stellifera* (A. Adams & Reeve [in A. Adams], 1848)	CASIZ167989	Hawaii	HM162703	HM162623	HM162529
*Bornella valdae* Pola, Rudman & Gosliner, 2009	CASIZ176832	South Africa	HM162706	HM162626	HM162532
*Crosslandia viridis* Eliot, 1902	CASIZ192342	Saudi Arabia	KP871637	KP871685	KP871661
*Doto amyra* Er. Marcus, 1961	CASIZ181213	USA: California	KJ486703	KJ486768	KJ486674
*Dendronotus dalli* Bergh, 1879	ZMMU:Op-295	Kamchatka	KM397001	KM397083	KM397094
*Dendronotus iris* J. G. Cooper, 1863	LACM 174194	USA: California	KX058082	GU339188	KX058110
*Dendronotus frondosus* (Ascanius, 1774)	ZMMU:Op-380	Norway	KM396976	KM397056	KM397111
*Dendronotus regius* Pola & Stout, 2008	CASIZ179493	Philippines	JN869451	JN869407	JN869430
*Dendronotus robustus* Verrill, 1870	ZMMU: Op-390	Barents Sea	KM396963	KM397045	KM397115
*Dermatobranchus* sp.1	CASIZ177375	Philippines	HM162698	HM162616	HM162522
*Dermatobranchus* sp.2	CASIZ176273	South Africa	HM162697	HM162609	HM162515
*Doridomorpha gardineri* Eliot, 1903	CASIZ178233	Malaysia	HM162695	HM162605	HM162511
*Doridoxa walteri* (Krause, 1892)	ZMMU:Op-721	Russia: Barents Sea	**MW139263**	**MW144285**	**MW158320**
*Doridoxa walteri* (Krause, 1892)	ZMMU:Op-722	Russia: Barents Sea	**MW139262**	**MW144284**	-
*Doridoxa “ingolfiana”* Bergh, 1899	CPIC01052	Canada: Newfoundland	KP871640	KP871688.	-
*Doto antarctica* Eliot, 1907	DANT1	Antarctica	KX274295	KX274324	KX274308
*Doto coronata (Gmelin*, *1791)*	BELUM Mn33135	UK	KJ486723	KJ486762	KJ486653
*Doto dunnei* Lemche, 1976	DDUN1	Mediterranean	KX274292	KX274318	KX274300
*Doto koenneckeri* Lemche, 1976	CASIZ178247	Portugal	HM162735	HM162658	HM162567
*Doto millbayana* Lemche, 1976	BELUM Mn33145	UK	KJ486726	KJ486759	KJ486660
*Duvaucelia lineata* (Alder & Hancock, 1848)	GNM8890	Sweden	MG934992	-	-
*Duvaucelia manicata* (Deshayes, 1853)	-	-	KY629602	KY629592	KY629606
*Duvaucelia nilsodhneri* (Marcus Ev., 1983)	Med1	Mediterranean	KY629600	KY629585	KY629611
*Duvaucelia nilsodhneri* (Marcus Ev., 1983)	Atl2	Atlantic	KY629597	KY629591	KY629605
*Duvaucelia* cf. *nilsodhneri* (Marcus Ev., 1983)	CASIZ 176218A	South Africa	KP153294	KP153261	KP153327
*Duvaucelia* cf. *nilsodhneri* (Marcus Ev., 1983)	CASIZ 176218B	South Africa	KP153295	KP153262	KP153328
*Duvaucelia* cf. *nilsodhneri* (Marcus Ev., 1983)	CASIZ 176218C	South Africa	KP153296	KP153263	KP153329
*Duvaucelia* cf. *nilsodhneri* (Marcus Ev., 1983)	CASIZ 176219	South Africa	HM162716	HM162641	HM162548
*Duvaucelia plebeia* (G. Johnston, 1828)	ZMMU:Op-572	Norway	KX788134	KX788122	-
*Duvaucelia plebeia* (G. Johnston, 1828)	-	North Sea	KR084473	-	-
*Duvaucelia plebeia* (G. Johnston, 1828)	-	North Sea	KR084842	-	-
*Duvaucelia plebeia* (G. Johnston, 1828)	-	North Sea	KR084538	-	-
*Duvaucelia plebeia* (G. Johnston, 1828)	-	Sweden	-	AJ223393	-
*Duvaucelia striata* (Haefelfinger, 1963)	BAU2696	Italy	LT596541	LT596543	LT615408
*Duvaucelia striata* (Haefelfinger, 1963)	BAU2695	Italy	LT596540	LT596542	LT615407
*Hancockia californica* MacFarland, 1923	CASIZ175722	Costa Rica	HM162702	HM162702	HM162527
*Hancockia californica* MacFarland, 1923	LACM 174934	Mexico	JN869452	JN869408	JN869433
*Hancockia uncinata (Hesse*, *1872)*	SRR3726694	United Kingdom	KX889735	MK100971	-
*Heterodoris robusta* Verrill & Emerton, 1882	ZMMU:Op-723	Canada and Greenland	**MW139261**	**MW144283**	**MW158318**
*Lomanotus* sp.1	CASIZ 177751	Philippines	JN869453	JN869409	JN869434
*Lomanotus* sp.2	LACM174962	Mexico	HM162715	HM162640	-
*Lomanotus vermiformis* Eliot, 1908	SRR3726706	Panama	KX889740	MK100978	-
*Lomanotus vermiformis* Eliot, 1908	CASIZ175963	Mexico	-	-	JN869435
*Luisella babai* (Schmekel, 1972)	MNCN15.05/53698	Spain	HQ616783	HQ616754	HQ616717
*Marianina rosea* (Pruvot-Fol, 1930)	CASIZ175746	Philippines	HM162733	HM162656	HM162565
*Marionia abrahamorum* F. V. Silva, Herrero-Barrencua, Pola & Cervera, 2019	MB28005053	Gulf of Guinea	MH892390	MH892386	MH892392
*Marionia arborescens* Bergh, 1890	CASIZ177578	Philippines	HM162722	HM162646	HM162554
*Marionia blainvillea* (Risso, 1818)	CASIZ176812	Portugal	HM162721	HM162645	HM162553
*Marionia blainvillea* (Risso, 1818)	isolate 2	-	KY629604	KY629593	KY629613
*Marionia distincta* Bergh, 1905	CASIZ173317	Philippines	HM162725	HM162648	HM162557
*Marionia elongoviridis* V. G. Smith & Gosliner, 2007	CASIZ173308	Philippines	HM162724	-	HM162556
*`Marionia gemmii* Almón, Pérez & Caballer, 2018	TRI0316	Spain: Galicia	KY584069	KY584068	-
*Marionia levis* Eliot, 1904	CASIZ192357A	Saudi Arabia	KP153284	KP153251	KP153317
*Marionia* sp.1	MB28005057	Spain: Gulf of Cadiz	MH892391	MH892389	MH892395
*Melibe leonina* (Gould, 1852)	SRR1950947	USA: CA	KX889741	KX889741	-
*Melibe leonina* (Gould, 1852)	-	USA: CA, Monterey	KP764764	KP764764	-
*Notobryon wardi* Odhner, 1936	CASIZ177540	Philippines	JN869454	JN869411	JN869437
*Notobryon* sp.	CASIZ 176363	South Africa	HM162713	HM162636	HM162543
*Notobryon thompsoni* Pola, Camacho-Garcia & Gosliner, 2012	CASIZ176362	South Africa	JN869456	JN869413	JN869439
*Pleurobranchaea meckeli* (Blainville, 1825)	-	Mediterranean Sea, Spain	FJ917499	FJ917439	EF133470
*Pleurobranchaea meckeli* (Blainville, 1825)	-	Spain: Gerona	AY345026	AY345026	-
*Pleurobranchus varians* Pease, 1860	CPIC00351	USA: Hawaii	KM521700	KM521597	KM521625
*Samla takashigei* Korshunova, Martynov, Bakken, Evertsen, Fletcher, Mudianta, Saito, Lundin, Schrödl & Picton, 2017	ZMMU:Op-530	Japan	MF523384	MF523463	MF523309
*Scyllaea pelagica* Linnaeus, 1758	CASIZ184317	Philippines	JN869459	JN869416	JN869443
*Scyllaea fulva* Quoy & Gaimard, 1824	SRR3726701	French Polynesia	KX889746	MK100991	-
*Tethys fimbria* Linnaeus, 1767	-	Spain: Tarragona	AY345035	AY345035	EF133468
*Tochuina gigantea* (Bergh, 1904)	ZMMU:Op-726	Canada:British Columbia	**MW139260**	**MW144282**	**MW158321**
*Tochuina gigantea* (Bergh, 1904)	BFHL-2218	USA: Washington	MH243006	-	-
*Tochuina nigritigris* (Valdés, Lundsten & N. G. Wilson, 2018)	LACM 3553	USA: CA	MH756138	MH756133	-
*Tritonia* cf. *antarctica* Pfeffer in Martens & Pfeffer, 1886	-	Antarctica, Ross Sea	GQ292052	-	-
*Tritonia challengeriana* Bergh, 1884	CASIZ171177	Atlantic Ocean: Bouvet Island	HM162718	HM162643	HM162550
*Tritonia challengeriana* Bergh, 1884	CASIZ189419	Atlantic Ocean: Falkland Islands	KP153310	KP153277	KP153343
*Tritonia exsulans* Bergh, 1894	ZMMU: Op-720	USA: Washington	**MW139259**	**MW144281**	-
*Tritonia exsulans* Bergh, 1894	-	Canada: Vancouver	KP764765	KP764765	-
*Tritonia exsulans* Bergh, 1894	LACM:2004–16.3	USA: California	-	GU339203	-
*Tritonia exsulans* Bergh, 1894	LACM:DISCO:4046	North Pacific Ocean	BOLD:AAW7932	-	-
*Tritonia exsulans* Bergh, 1894	-	USA: Washington	GQ292050	-	-
*Tritonia festiva* (Stearns, 1873)	CASIZ186478	USA: CA	KP153291	KP153258	-
*Tritonia festiva* (Stearns, 1873)	SRR1950941	USA: CA	KX889748	MK100994	-
*Tritonia festiva* (Stearns, 1873)	-	USA, Washington	GQ292051	-	-
*Tritonia festiva* (Stearns, 1873)	CASIZ174491	USA: Oregon	HM162719	-	HM162551
*Tritonia festiva* (Stearns, 1873)	CASIZ173748	USA: CA	-	KP153270	KP153336
*Tritonia hombergii* Cuvier, 1803	ZMMU: Op-724	Norway	**MW139258**	**MW144280**	**MW158319**
*Tritonia hombergii* Cuvier, 1803	MT09685	North Sea	KR084797	-	-
*Tritonia hombergii* Cuvier, 1803	GNM: Gastr8763V	Sweden	MG934917	-	-
*Tritonia hombergii* Cuvier, 1803	GNM: Gastr 8761V	Sweden	MG935087	-	-
*Tritonia tetraquetra* (Pallas, 1788)	SIO-BIC M12395	USA	MH756139	MH756134	MH756145
*Tritonia tetraquetra* (Pallas, 1788)	ZMMU:Op-719	Russia: Kamchatka	**MW139257**	**MW144279**	-
*Tritonia tetraquetra* (Pallas, 1788)	ZMMU:Op-725	Canada: British Columbia	**MW139256**	**MW144278**	-
*Tritonia* cf. *psoloides* Aurivillius, 1887	CASIZ181055	Bering sea	KP153304	KP153271	KP153337
*Tritonicula bayeri* (Marcus & Marcus, 1967)	CPIC01540	Panama	-	MN162697	MN162696
*Tritonicula hamnerorum* (Gosliner & Ghiselin, 1987)	CASIZ181095	Bermuda	KP153292	KP153259	KP153325
*Tritonicula hamnerorum* (Gosliner & Ghiselin, 1987)	CASIZ181090	Bermuda	KP153293	KP153260	KP153326
*Tritonicula myrakeenae* (Bertsch & Mozqueira, 1986)	CCDB 24004 B06	California	BOLD:ADW5537	-	-
*Tritonicula pickensi* (Ev. Marcus & Er. Marcus, 1967)	CASIZ175718	Costa Rica	-	HM162642	HM162549
*Tritoniella belli* Eliot, 1907	-	Ross Sea	GQ292056	-	-
*Tritoniella belli* Eliot, 1907	N31D	Antarctica	GU227111	GU227002	-
*Tritoniopsis elegans* (Audouin, 1826)	CASIZ69928	Japan	KP153314	KP153281	KP153347
*Tritoniopsis frydis* Er. Marcus & Ev. Marcus, 1970	CASIZ181156	Bermuda	KP153311	KP153278	KP153344
*Tritoniopsis frydis* Er. Marcus & Ev. Marcus, 1970	SRR1950954	USA: Florida	KX889749	MK088234	-
*Tritoniopsis* sp.	CASIZ191453A	Papua New Guinea	KP153312	KP153279	KP153345
*Tritoniopsis* sp.	CASIZ191453B	Papua New Guinea	KP153313	KP153280	KP153346

### Nomenclatural acts

The electronic edition of this article conforms to the requirements of the amended International Code of Zoological Nomenclature, and hence the new names contained herein are available under that Code from the electronic edition of this article. This published work and the nomenclatural acts it contains have been registered in ZooBank, the online registration system for the ICZN. The ZooBank LSIDs (Life Science Identifiers) can be resolved and the associated information viewed through any standard web browser by appending the LSID to the prefix "http://zoobank.org/". The LSID for this publication is: urn:lsid:zoobank.org:pub: 0D2A82C2-AA25-4B93-96A8-7158D56F6477. The electronic edition of this work was published in a journal with an ISSN and has been archived and is available from the following digital repositories: PubMed Central, LOCKSS.

## Results and discussion

### Molecular analysis

The phylogenetic analysis was performed using sixty-one specimens of the family Tritoniidae, and forty-five related taxa. The dataset consisted of two hundred and fifty eight nucleotide sequences. The HKY + I + G model was chosen for the 16S; the GTR + I + G model was chosen for COI, H3, and for the combined dataset. The resulting combined tree provided better resolution than COI, 16S, or H3 separately (Fig 1A, S1 Fig). Bayesian Inference (BI) and Maximum Likelihood (ML) analyses based on the combined dataset for the mitochondrial COI and 16S, and the nuclear H3 genes yielded similar results (Fig 1A).

The molecular phylogenetic analyses support the existence of the several main clades within the family Tritoniidae. *“Tritonia” manicata*, *“T*.*” lineata*, *“T*.*”striata*, *“T*.*” plebeia*, and *“T*.*” nilsodhneri* show close evolutionary relationships and clustered together with the maximal support (PP = 1, BS = 100) in a separate clade that attributes here as *Duvaucelia* (restricted) clade. *“Tritonia” bayeri*, *“T*.*” myrakeenae*, *“T*.*” pickensi*, and *“T*.*” hamnerorum* form separate clade (PP = 1, BS = 76) that attributes here as *Tritonicula* gen. nov. clade. All another *Tritonia* species clustered together in a clade that is sister to *Duvaucelia* (restricted) clade. All five *Tritonia exsulans*, five *T*. *festiva*, three *T*. *tetraquetra* and *T*. cf. *psoloides* clustered together in separate clade with high support (PP = 1, BS = 99), that branched to three clades. The first clade (PP = 1, BS = 99) includes *T*. *tetraquetra* and *T*. cf. *psoloides* is sister to the clade (PP = 1, BS = 92) divided into two subclades: *T*. *exsulans* and *T*. *festiva*. The ABGD analysis of the *Tritonia* sensu lato COI data set run with two different models revealed fifteen potential species, include *T*. *tetraquetra*, *T*. *exsulans*, *T*. *festiva*, *T*. cf. *psoloides*, and some others (see Fig 1A). Minimum uncorrected p-distances of the COI marker which separate *T*. *tetraquetra* from *T*. *exsulans*, *T*. *festiva*, and *T*. cf. *psoloides* are 10.96%, 11.4%, and 7.5% respectively. Minimum uncorrected p-distances of the COI marker which separate *T*. *exsulans* from *T*. *festiva*, and *T*. cf. *psoloides* are 9.09% and 10.88% respectively. Minimum uncorrected p-distances of the COI marker between *T*. *festiva* and *T*. cf. *psoloides* are 11.63% (Table 2). Results the ABGD analysis from recursive partitions show two potential species in group*“T*.*” nilsodhneri* from two different regions, Europe and South Africa, whereas results from initial partitions show only one. The specimens previously identified as *“T*. *nilsodhneri”* from South Africa therefore likely belong to an undescribed species, pending further revision. Publicly available COI sequences *T*. *challengeriana* and *T*. *antarctica* were revealed by ABGD analysis as two different potential species. *Tritonia antarctica* therefore needs a separate study, including molecular, morphological, and geographical data.

**Table 2:** Intraspecific (highlighted in bold) and interspecific uncorrected p-distances (range, %) for COI marker in species of genera *Tritonia*, *Tochuina*, *and Doridoxa*.

	*Tritonia exsulans*	*Tritonia festiva*	*Tritonia tetraquetra*	*Tritonia* cf. *psoloides*	*Tritonia hombergii*	*Tritonia challengeriana*	*Tritonia* cf. *antarctica*	*Tochuina gigantea*	*Tochuina nigritigris*	*Doridoxa walteri*	*Doridoxa “ingolfiana”*
*Tritonia exsulans*	**0–1.01**	9.09–10.46	10.96–11.80	10.88–11.09	16.64–17.35	15.83–16.72	16.28–16.49	18.11–18.72	20.24–20.57	21.16–21.75	20.55–21.08
*Tritonia festiva*	9.09–10.46	**0.61–2.81**	11.4–12.94	11.63–12.2	15.0–16.89	15.37–16.88	15.22–16.91	19.17–19.79	18.35–19.94	18.84–20.55	18.18–19.94
*Tritonia tetraquetra*	10.96–11.80	11.4–12.94	**0–0.46**	7.5–8.07	15.04–16.13	15.37–15.76	15.86–16.28	18.57–19.18	19.48–19.94	19.63–19.79	19.33–19.48
*Tritonia* cf. *psoloides*	10.88–11.09	11.63–12.2	7.5–8.07	**-**	15.2–15.76	15.2–15.38	15.51	17.64–17.82	17.82	18.57	18.2
*Tritonia hombergii*	16.64–17.35	15.0–16.89	15.04–16.13	15.2–15.76	**0–0.64**	18.72–20.38	17.76–18.39	17.44–18.42	18.82–19.79	20.8–21.18	20.16–20.55
*Tritonia challengeriana*	15.83–16.72	15.37–16.88	15.37–15.76	15.2–15.38	18.72–20.38	**2.71**	13.32–13.74	17.83–18.42	17.99–19.03	20.55–21.82	20.7–21.82
*Tritonia* cf. *antarctica*	16.28–16.49	15.22–16.91	15.86–16.28	15.51	17.76–18.39	13.32–13.74	**-**	18.39–18.6	18.6	20.08–20.3	19.66
*Tochuina gigantea*	18.11–18.72	19.17–19.79	18.57–19.18	17.64–17.82	17.44–18.42	17.83–18.42	18.39–18.6	**0.15**	15.83–15.98	20.7–21.0	20.24–20.4
*Tochuina nigritigris*	20.24–20.57	18.35–19.94	19.48–19.94	17.82	18.82–19.79	17.99–19.03	18.6	15.83–15.98	**-**	21.61–21.77	20.85
*Doridoxa walteri*	21.16–21.75	18.84–20.55	19.63–19.79	18.57	20.8–21.18	20.55–21.82	20.08–20.3	20.7–21.0	21.61–21.77	**0.15**	0.76–0.91
*Doridoxa “ingolfiana”*	20.55–21.08	18.18–19.94	19.33–19.48	18.2	20.16–20.55	20.7–21.82	19.66	20.24–20.4	20.85	0.76–0.91	**-**

Highly supported clade *Tritoniopsis* (PP = 1, BS = 99) is sister to *Marianina rosea* (PP = 1, BS = 76). All *Marionia* clustered together (PP = 1, BS = 100) in a separate clade. Clade *Tochuina* is sister (PP = 1, BS = 77) to *Tritoniella belli* clade. It is important to note that *“Tritonia” nigritigris* has the closest position to the clade *Tochuina gigantea* and clustered together with the maximal support (PP = 1, BS = 100). The ABGD analysis of the COI data set revealed two potential species: *“Tritonia” nigritigris* and *Tochuina gigantea*. Minimum uncorrected p-distances of the COI marker between *Tochuina gigantea* and *“Tritonia” nigritigris* are 15.83% (Table 2).

*Heterodoris robusta* and *Doridomorpha gardineri* clustered in two distinct and separated sister clades (PP = 1, BS = 97) that formed the sister group (PP = 0.99, BS = 70) to the *Doridoxa* clade. Two *Doridoxa walteri* and *D*. *ingolfiana* clustered together in clade with maximal support (PP = 1, BS = 100). ABGD analysis of the COI data set revealed one potential species, combined *D*. *walteri* and *D*. *ingolfiana*. Uncorrected p-distances of the COI marker within the *Doridoxa walteri* and *D*. *ingolfiana* range from 0.15% to 0.91%, indicating that they are the same species (Table 2).

### Analysis of taxonomic history of the North Pacific model species tritoniids

The systematics of the North Pacific species of the family Tritoniidae has a long history.

Peter Simon Pallas [38] described the first northern Pacific tritoniid under the name *Limax tetraquetra* (now *Tritonia tetraquetra*) from the North Kuril Islands (Fig 2A and 2B). Pallas did not collect himself the materials that he published in 1788. The collection and descriptions were performed by Georg Wilhelm Steller. He was a naturalist who participated in the Vitus Bering’s Second Kamchatka Expedition [39]. Steller had a short trip to three northernmost Kuril Islands in May-June 1743 [40]. Detailed descriptions of invertebrates (including *Tritonia tetraquetra*), fishes and the sea-otter were made by Steller during Kuril and South Kamchatka voyages. After Steller’s premature death some of his data were published by Pallas [39, 41].

Only after a century, *Limax tetraquetra* was attributed to the genus *Tritonia* by Bergh in 1879 [42] and this species name was applied to a specimen from Unalashka Island. On the first line of the description of *T*. *tetraquetra* Bergh ([42]: p. 98) noted that “this species was detected by Pallas….”. However, an incorrect comparison with the Pallas’s description is given in a footnote: “the form of the mandibulae is rather similar to the figure in Pallas ([38]: Fig 22)” ([42]: p. 102). The jaws (mandibulae) of *T*. *tetraquetra* as described by Bergh [42: p. 102] considerably different from the jaws shape in the original description of *L*. *tetraquetra* by Pallas ([38]: Fig 22) (Fig 2B). No any other evidence for the similarity of Unalashka’s and Kuril’s specimens was given. Later on, Bergh (1904, [43]) described a new species *Tritonia gigantea* based on a collection from Unalaska Island too. *Tritonia gigantea* description is a nearly identical to the *T*. *tetraquetra* description from Bergh (1879, [42]).

According to the original description of *Limax tetraquetra* by Pallas ([38]: 237–239) this species possesses bilobed oral veil with distinct processes, a high body without traces of lateral edges of notum (Fig 2A) and elongate-oval jaws (Fig 2B). To contrary, *T*. *tetraquetra* and *T*. *gigantea* described by Bergh in 1879 and 1904 [42, 43] have non-bilobed oral veil without distinct appendages (only with tubercles), low body with strongly projected lateral sides of notum (Fig 3A and 3B) and nearly square short jaws (Fig 3F). Thus Bergh in 1879 [42] incorrectly attributed Pallas’s *Limax tetraquetra* to the genus *Tritonia*. However, all subsequent authors [15, 22, 44–48] followed Bergh’s decision. Odhner [23] in a review of the family Tritoniidae, proposed a new genus *Tochuina* Odhner, 1963 for *Tritonia tetraquetra* sensu Bergh, 1879 in a combination “*Tochuina tetraquetra*”. *Tritonia gigantea* was considered as a synonym of “*Tochuina tetraquetra*” [47, 48].

Unlike the genus *Tochuina* (Fig 3), the outlined above characters of the real *Limax tetraquetra* shared much in common with the genus *Tritonia* (Fig 2). The inappropriate application of the characters of the genus *Tochuina* to *Limax tetraquetra* persisted until recently. Afterwards, *Limax tetraquetra* was reassigned as *Tritonia tetraquetra*, and type species of the genus *Tochuina* was re-designated to *Tochuina gigantea* [17, 18]. In the present study we for the first time present morphological data on the *Tochuina gigantea* holotype from the Natural History Museum of Denmark (ZMUK GAS-2011, not suitable for molecular research) (Fig 3A–3D) and molecular and morphological data for the recently collected specimen *Tochuina gigantea* from British Columbia (Fig 3J–3R). Results clearly confirm that *T*. *gigantea* displays morphological characters that were incorrectly assigned to *Limax tetraquetra* by Bergh [42] and Odhner [23]. “*Tritonia*” *gigantea* (original binomen in Bergh, 1904, [43]) thus belongs to the genus *Tochuina* and according to the present analysis shows a separate phylogenetic lineage within the family Tritoniidae, distantly related to the genus *Tritonia* (see Fig 1A and 1B and a taxonomic synopsis below). “*Limax” tetraquetra* (original binomen in Pallas, 1788 [38]) instead belongs to a clade closely related the type species of the genus *Tritonia*, *T*. *hombergii* Cuvier, 1803 (Fig 1A).

Only a single large-sized species of *Tritonia* was previously recognized from the shallow waters of the NW Pacific and Kuril Islands, the type locality of *Tritonia tetraquetra* [17, 18, 49]. It is *Tritonia diomedea* Bergh, 1894. This species was described by Bergh (1894 [50]) based on deep-sea specimens from California and the Shumagin Islands. In the same publication Bergh [50] also described a shallow water species from California under the name *Tritonia exsulans* (Fig 4). This species was recognized in a few works [45–47] but then *T*. *exsulans* was commonly considered as a synonym of *T*. *diomedea* [15]. This decision was made because of a putative absence of distinguishing characters between *T*. *diomedea* and *T*. *exsulans*. Three more species were described from NW Pacific, namely *Tritonia psoloides* Aurivillius, 1887 [51], *T*. *septemtrionalis* (Baba, 1937) [52], and *T*. *primorjensis* Minichev, 1971 [53] (Fig 5). One more species, *T*. *gilberti* (MacFarland, 1966) [47], was described from NE Pacific. All of them were considered as synonym of *T*. *diomedea* [15] without detailed analysis. This decision has persisted in the literature on the North Pacific fauna until recently, including colour guides, ecological and neurobiological experimental works.

Among the large North Pacific tritoniids, species in *Tritonia* and *Tochuina* genera were most widely used as neuroscience model systems. Majority of the neurobiological research in *Tritonia* were done both on NE and NW coasts of the North Pacific (see list of works in [15]), using the species name “*T*. *diomedea* Bergh, 1894”. “*Tochuina tetraquetra* (Pallas, 1788)” sensu Bergh 1879, not Pallas 1788, has been also studied for neural basis of behaviour [8, 13].

### Morphological and molecular delimitations of *Tritonia* model species from the northern Pacific

Species delimitation based on morphological characters between *T*. *tetraquetra* sensu Pallas not Bergh (previously identified as “*T*. *diomedea”*) and *T*. *exsulans* was uncertain [47, 48] or considered as not reliable [15]. In the present study robust molecular differences (Fig 1A), and considerable differences in fine morphology of the masticatory edges of the jaws between real *Tritonia tetraquetra* and *T*. *exsulans* were revealed (Figs 2I–2K, 4G and 4H). Bergh in 1894 ([50]: plate 4, Fig 2) clearly figured smaller irregular and polygonal elements for *Tritonia diomedea* that transit to larger tablet-like narrow endings of the tightly packed long bristle-like elements on the masticatory edge of the jaws (Fig 2V). These elements can be also described as long, narrow, flattened spines (Fig 2I–2K), but not as massive cones (Fig 4G and 4H). The details of the masticatory edge of *T*. *diomedea* readily differ from distinct conical elements throughout entire masticatory edge, without table-like endings in *Tritonia exsulans* (Fig 4G, 4H and 4M). In the original description of *T*. *diomedea* “4–5 quincunx rows of small cones gradually change into a number of fine tablets on the outside” are mentioned (Bergh, 1894 [50]: p. 148) (Fig 2V). This description well matches to our scanning electron microscopic data for *T*. *tetraquetra*: initially polygonal and irregular conical rows of elements (Fig 2I–2K) ended up with long, narrow plates. This structure after dissection of the masticatory edge turned to be tightly packed clusters of bristle-like, elongated elements (Fig 2H and 2I). The polygonal elements (with missing bristles, Fig 2K) are figured also in [19] for a *Tritonia* sp. from the deep waters (587–610 m) off Oregon. The mentioned *Tritonia* sp. molecularly matches to our confirmed specimens of *T*. *tetraquetra* from shallow waters of Kamchatka and British Columbia (Fig 1A). These important results are the first confirmation that *Tritonia tetraquetra* has a very broad geographic and bathymetric range, as it was suggested earlier [17, 18]. Instead, *T*. *exsulans* (Bergh, 1894 [50]: plate 3, Fig 11) (Fig 4M) has the massive distinct conical elements embedded into masticatory edge and it is impossible to reveal any elongate bristle-like elements after dissection of the edge (Fig 4G and 4H). Bergh [50] also clearly figured these elements in *T*. *exsulans* ([50]: plate 3, Fig 11) and described it (compare to *T*. *diomedea*) in a different way: “10–12 rows of polygonal flat plates, of which those of the 3–4 innermost rows forming stronger, lower oblique cones” ([50]: 151) (Fig 4M). McDonald in 1983 [15] dismissed taxonomic importance of these differences using previously published light microscopic data. In the present study these differences were confirmed using scanning electron microscopic data (Figs 2G–2K and 4F–4H). The patterns of the masticatory edges elements are stable and easily identifiable in both first descriptions of *T*. *diomedea* and *T*. *exsulans* respectively [50]. Thus, *Tritonia tetraquetra* (and its junior synonym *T*. *diomedea*) is a different from *T*. *exsulans* species.

According to the integrative molecular and morphological data *T*. *tetraquetra* and *T*. *exsulans* definitely represent two distinct separate species (Figs 1A, 2 and 4). There is only available syntype of *T*. *diomedea* in the Natural History Museum of Denmark (ZMUK, GAS-2034) that originated from a location in California (Fig 2Q–2T). This type specimen comes from essentially the same depth (676 m vs 587–610 m) with a molecularly confirmed specimen of *T*. *tetraquetra* from geographically close to the California waters of Oregon (“*Tritonia sp*.” in [19]: 410; present study, Figs 1A and 6). All reliable records of *T*. *exsulans* [47, 50, present study] do not exceed the depth about 100 meters. Instead, the confirmed *T*. *tetraquetra* (Figs 1A and 6) has a very broad bathymetric range, from shallow waters (5–10 m) up to at least 610 m depth. Another syntype specimen of *T*. *diomedea* mentioned in Bergh [50] from deep waters of Alaska (the Shumagin Islands) is lost [54]. The saved syntype of *T*. *diomedea* (ZMUK, GAS-2034) from deep waters of California is designated here as lectotype of *T*. *diomedea* (Fig 2Q–2S) to avoid a potential confusion of “*T*. *diomedea”* in original sense of Bergh [50] with some potential hidden molecular lineages within *T*. *tetraquetra*.

Based on the present analysis we therefore able to reveal a clear presence of two species in the waters of NE Pacific that formerly were known under the name of “*T*. *diomedea*”: *T*. *tetraquetra* (= *T*. *diomedea*) and *T*. *exsulans* (Figs 1A and 6). *T*. *exsulans* is more warm water adapted species because distributes to the southern California and never reliably recorded from the colder NW Pacific. Herein the molecular data throughout all the geographic range of *T*. *exsulans* from California to British Columbia applied (Fig 6). Therefore it is reliably estimated that *T*. *exsulans* was also used for neurobiological experiments, commonly hold at the Friday Harbor Laboratories [8, 20]. Because in the region of the Friday Harbor *T*. *tetraquetra* and *T*. *exsulans* can co-occur (Fig 6), both species could be used as model systems for neurobiological and related research at NE Pacific coast.

Another known nominative species of *Tritonia* from NW Pacific, *T*. *septemtrionalis* (Baba, 1937) [52] was described from the relatively shallow waters (82 m) of the Okhotsk Sea near Kamchatka region. According to the morphological data, depicted in Baba ([52]: Fig 1F), *T*. *septemtrionalis* shows a not dissected massive of the long bristles on masticatory edges of the jaws. According to morphological and distributional data *T*. *septemtrionalis* can be therefore considered as a junior synonym of *T*. *tetraquetra*. Present molecular data (Fig 1A) confirmed only true *T*. *tetraquetra* in the shallow waters of the neighbouring Kamchatka region.

Further molecular lineages, closely related to real *T*. *tetraquetra* can be potentially revealed in NW Pacific. For example, in the present study we revealed the publicly available sequence from the Bering Sea that somewhat diverges from *T*. *tetraquetra*, but definitely belongs to the same clade (Figs 1A and 6). Because the only single sequence is available on GenBank and we have no possibility conduct morphological analysis, we can not absolutely confidently confirm this species. However, potentially it belongs to *T*. *psoloides* Aurivillius, 1887 [51], a species of *Tritonia* that was described from the Bering Sea. The type specimen of *T*. *psoloides* comes from the depth about 140 m, whereas potential *T*. *psoloides* specimen comes from 402 m depth (Fig 6). Therefore the same species may habitat in the same locality and similar bathymetric range. The type specimen of *T*. *psoloides* is in the Swedish Museum of Natural History and was available for the present study (Fig 5A–5E). The external appearance (Fig 5A–5C), radular teeth (Fig 5F) and bathymetric distribution of *T*. *psoloides* ([51]: p. 373, plate 13, Fig 20; present study) are generally consistent with *T*. *tetraquetra*. Therefore, *T*. *psoloides* is an available name for that potentially hidden lineage (Fig 1A) close to *T*. *tetraquetra* in the Bering Sea. We therefore do not consider *T*. *psoloides* as a synonym of *T*. *tetraquetra* and preliminary assign a specimen from the Bering Sea with available molecular data to *T*. *cf*. *psoloides* (Figs 1A and 6) pending a further study.

There is one more species from NW Pacific, *Tritonia primorjensis* Minichev, 1971, described from the Sea of Japan (Fig 5G–5V). Molecular data were not available for this species due to formalin fixation, but morphologically *T*. *primorjensis* is consistent with *T*. *tetraquetra* in absence of the white lines and presence of bristle-like elements on the masticatory edges of the jaws ([53]; present study, Fig 5M and 5N). Minichev ([53]: p. 282) thus correctly indicated the presence of the clusters of narrow masticatory elements (Fig 5S and 5T), but incorrectly estimated its differences from *T*. *tetraquetra* (= *T*. *diomedea*). We specially studied the morphology of the jaws in potential *T*. *primorjensis* from the Sea of Japan. The specimens of *T*. *primorjensis* were collected in the Peter the Great Bay (the Sea of Japan) and used by Soviet neurophysiologists in the 1960s – 1980s for experimental works. We confirm here the basic similarity of *T*. *primorjensis* to *T*. *tetraquetra* using our scanning electron microscopic data (Figs 2I, 2J, 5M and 5N) and correctness of the Minichev’s description of the masticatory edge of the jaws with long thin spines (Fig 5T). However, *T*. *primorjensis* differs from *T*. *tetraquetra* by longer spines at the masticatory edges (Figs 2I, 2J, 5M and 5N) and more intensive reddish-orange body colouration compare to the orange (with a slight reddish tinge) or yellow *T*. *tetraquetra* from British Columbia and Kamchatka. *T*. *primorjensis* potentially represents a further hidden lineage close to *T*. *tetraquetra*. Therefore *T*. *primorjensis* not included into synonymy of *T*. *tetraquetra* (a taxonomic diagnosis see below). The smaller difference in colouration is consistent with the modern agenda of the fine-scale species differentiation, but needs an additional testing in this *Tritonia* species complex. Thus, Russian neurophysiologists worked exclusively on a species that closely related to *T*. *tetraquetra*, but definitely not on *T*. *exsulans*, which does not occur in NW Pacific (Figs 1A and 6). Renewed taxonomic diagnoses for *T*. *tetraquetra* and *T*. *exsulans* are provided below.

### Taxonomic diagnoses of *Tritonia tetraquetra* and *T*. *exsulans*

*Tritonia tetraquetra* (Pallas, 1788)

Fig 2

*Limax tetraquetra* Pallas, 1788 [38]: 237–239, pl. 5, Fig 22

*Tritonia tetraquetra* (in original sense of Pallas, 1788)–Martynov, 2006a [17]: 280, pl. 134 F–H; Martynov, 2006b [18]: 69

*Tritonia diomedea* Bergh, 1894 [50]: 146–150, pl. 2, Figs 10, 11, pl. 3, Figs 6–10, pl. 4, Figs 1–5

*Tritonia diomedia*–O’Donoghue, 1921 [55]: 151–152, pl. 7, Figs 1–3; Volodchenko, 1955 [56]: 249, pl. 48, Fig 3; Veprintsev et al., 1964 [6]: 327–336, Figs 1, 2; Borovyagin and Sakharov, 1968 [7]: 3 (a mistake in spelling (“*diomedia*” vs. correct “*diomedea*” appeared after O’Donoghue (1921) [55] in numerous further publications, the list in McDonald, 1983 [15])

*Tritonia diomedea* sensu Thompson, 1971 [47] and McDonald, 1983 [15] and auct.–partim. (mixture with *T*. *exsulans*)

*Duvaucelia* (*Duvaucelia*) *septemtrionalis* Baba, 1937 [52]: 391–392, text Figs 1a–1e

Non *Tritonia tetraquetra* sensu Bergh, 1879 [42] and auct. (= *Tochuina tetraquetra* sensu Odhner, 1963 [23])

Diagnosis. Colouration commonly yellow-orange to dark orange with a slight reddish tinge. No white lines along edges of lateral side between dorsolateral appendages and on oral veil. White line on edge of foot absent. Masticatory processes of jaws with clusters of bristle-like thin elongate plates. Radular formula ca. 40–73 x 150–50.1.50–150. Seminal receptacle small, oval, with long thin stalk and large wide, rounded bag-like base. Copulative organ massive, folded. Body length up to 300 mm. Confirmed bathymetric range about 1–700 m (potentially to about 1000 m).

Remarks. Type materials for *T*. *tetraquetra* are not traceable [17]. Neotype for *T*. *tetraquetra* is designated here (NW Pacific, Kamchatka Peninsula, ca. 10 m depth, stones, 11.08.2008, coll. Tatiana Korshunova, Alexander Martynov (ZMMU Op-719), live length 120 mm, preserved length 80 mm. Sources of the morphological data used for the diagnosis are [17, 38, 50] and present study (Fig 2C–2S). Details of taxonomic and phylogenetic position of *T*. *tetraquetra* and differences from related species see above.

***Tritonia exsulans* Bergh, 1894, reinstated**

Fig 4

*Tritonia exsulans* Bergh, 1894 [50]: 150–152, pl. 3, Figs 11–12, pl. 4, Fig 6; O'Donoghue, 1921 [55]: 152–154, pl. 7, Figs 4–6; MacFarland 1966 [47]: 226–235, pl. 30, Figs 9, 10, pl. 39, Fig 7, pl. 43, Figs 20–26; pl. 44, Figs 3, 4; pl. 45, Figs 9–13.

?*Duvaucelia gilberti* MacFarland, 1966 [47]: 223, 224, 235–243, pl. 30, Figs 1–2, pl. 43, Figs 27–36, pl. 44, Fig 5, pl. 45, Fig 6.

Non *Tritonia exsulans* sensu Thompson, 1971 [48] (mixture with *Tritonia tetraquetra*)

Non *Tritonia exsulans* sensu McDonald, 1983 [15] and auct. (incorrect synonymy with *T*. *tetraquetra*)

Non *Tritonia exsulans* sensu Baba, 1937 [57] (uncertain species attribution, possible *T*. *tetraquetra*)

Diagnosis. Colouration commonly pinkish to reddish salmon. White lines along edges of lateral side between dorsolateral appendages and on oral veil present. White line on edge of foot present. Masticatory processes of jaws with oval to conical strong elements, no thin clusters of bristle-like elements. Radular formula ca. 39–55 x 64–82.1.82–64. Seminal receptacle relatively large with long stalk and apparently with bag-like base. Copulative organ elongate with a circular fold. Body up to 200 mm. Confirmed bathymetric range about 5–100 m.

Remarks. Type materials for *T*. *exsulans* are not traceable (ZMUK type collection [54]). Neotype is designated here (NE Pacific, Port Orchard, Rich Passage, Washington, USA, 9.1 m depth, stones, 29.04.2017, coll. Karin Fletcher (ZMMU Op-720, live length 20 mm, preserved length 13 mm). Sources of the morphological data used for the diagnosis are [47, 50, 55] and present study (Fig 4A–4L). *Duvaucelia gilberti* (currently accepted as *Tritonia gilberti*) was described from the same geographic area with *T*. *exsulans* and MacFarland mentioned that in both species “The color is similar” ([47]: p. 242). The apparent main difference between *T*. *exsulans* and *T*. *gilberti* is in morphology of the copulative apparatus ([47]: p. 243), but listed differences are uncertain because indicated presence of variously expressed circular folds in both species. MacFarland [47] also found a minute armature in *T*. *exsulans* copulative organ, but this was not indicated in the original description of *T*. *exsulans* [42] and was not confirmed by further studies [48]. The presence of a widened base of the receptaculum seminis did not figured by MacFarland for *T*. *exsulans*, but indicated for *T*. *gilberti* ([47]: plate 44, 5). Therefore, with some reservation *T*. *gilberti* is considered here as a junior synonym of *T*. *exsulans*. Uncorrected p-distances values of the COI marker within the *T*. *exsulans* group range from 0 to 1.01% (Table 2), indicating low heterogeneity within all available *T*. *exsulans* molecular data on a broad geographic range from California to British Columbia.

### Origin of the generic name *Tochuina* and the role of *Tritonia tetraquetra* in human culture: A clarification and refuting

In the beginning of the original description of *Limax tetraquetra* from the northern Kuril Islands, Pallas ([38]: 237) indicated that “…ubi crudum coctumque edunt et *Tochui* appellant incolae.” (= [this species] was eaten as raw or cooked by local inhabitants). This short indication has been repeated as evidence that Ainu people (major inhabitants of the Kuril Islands at that time) was eating *T*. *tetraquetra* [22, 42, 58]. Incorrect subsequent spelling “*tochni*” for the Ainu word (originally spelled by Pallas as “*tochui*”) was also appeared in the listed works. Odhner [23] for the first time after Pallas [38] used original spelling *tochui* to name newly proposed genus *Tochuina* Odhner, 1963. Because of a long term confusion over real identity the Pallas’s original name *Limax tetraquetra* and misidentify it with the different tritoniid *Tochuina gigantea* [43] (see details above) the potential including of a large tritoniid into human diet was incorrectly attributed not to *Tritonia tetraquetra*, but to *Tochuina gigantea* [22, 42, 58]. The clarification of this issue is necessary to resolve persisted taxonomic confusion between *Tritonia* and *Tochuina* and this is therefore important part of the present study. The details are provided below.

Ainu were indigenous people of the Kuril Islands having their highly isolated language and unusual for Northeastern Asian ethnic groups external appearance [59, 60]. Fishing and hunting of marine mammals were important for the Ainu [61]. Besides, they collected a number of marine invertebrates for food. Several particular names for shelled molluscs have been attested for the Ainu language [62]. However, majority of nudibranchs including tritoniids, possess a strong chemical-based defense system [63]. Therefore proposals for an edible *Tritonia* (“*Tochuina*”) initially appeared as unrealistic. Pallas in the first description of *T*. *tetraquetra* [38] mentioned the vernacular Ainu name “*tochui*”. We need to investigate is that name was really used in Ainu language for nudibranch molluscs, or this is a case of a subsequent incorrect usage. For this purposes we used several existed Ainu dictionaries, including a compilative Dobrotvorsky dictionary [62] and a special dictionary with a comprehensive list of the Ainu names for animals [64]. The dictionary compiled by M.M. Dobrotvorsky encompasses words from many sources, including old Japanese and Russian sources and his own lexical data from the Sakhalin region [62]. Notably, in [62] we were able to identify at least two Ainu words which are considerable similar to the mentioned in Pallas [38] word *tochui*. These words are *totsui* and *togoi* [62]. The word *totsui* is indicated as “a molluscous animal”, but without mentioning of a particular species. Another potential word, *togoi* defined as “as a sea inhabitant with two teats; Ainu eat its viscera…”, with an addition “…a soft-bodied animal?[sic]” ([62]: p. 326). Such features can be reliably associated with two siphons of solitary ascidians, but not with molluscs. For instance, this can be attributed to a common North Pacific ascidian species *Halocynthia aurantium* (Pallas, 1788). *H*. *aurantium* was described (based on materials from Steller), remarkably, in the same publication of Pallas ([38]: pp. 246–247), a few pages after description of *T*. *tetraquetra*. *Halocynthia aurantium* shares with *T*. *tetraquetra* similar orange colouration and Steller definitely collected ascidian *H*. *aurantium* and nudibranch *T*. *tetraquetra* during the same expedition to the northern Kuril Islands. Thus, Steller could be informed by Ainu people with a vernacular ascidian name (similar to *totsui*/*togoi*) because nudibranch *T*. *tetraquetra* has the same colour and after taken out of water was similar to ascidian *H*. *aurantium*. It is also possible that a subsequent confusion arose after Steller applied the indigenous name for ascidian or nudibranch in his diaries. Ascidian *H*. *aurantium* is still used for food in the neighbouring Japanese Islands and available on the modern Japanese fish markets [65], which was confirmed during our recent expeditions to Japan.

Furthermore, a word similar in spelling to *totsui* and *tochui* (トツ°イ in katakana) is recorded in a comprehensive Ainu dictionary of the animal names [64] precisely for ascidians, and in a modern Japanese source [66] also for an ascidian species, *Halocynthia roretzi* (Drasche, 1884). Importantly, in XIX century the chordate ascidians still were incorrectly assigned to “molluscs”. That explain the indication of “molluscous or soft-bodied animals” for both *togoi* and *totsui* ([62]: pp. 326, 334). Thus, similarity of both Ainu words mentioned in the Dobrotvorsky’s dictionary as well as *totsui* in the Japanese sources [64, 66] to the Ainu word *tochui* indicated in the Pallas’s original description of *T*. *tetraquetra* can not be considered as an occasional one. Differences in spelling *tochui*/*totsui*/*togoi* can be potentially explained by dialectal differences, previously attested for the Kuril, Sakhalin and Hokkaido Ainu language [67] or by peculiarities of the Latin transliteration by Steller (or subsequently by Pallas) of an original spoken Ainu word. The Ainu dialect of the North Kuril Islands (type locality of *T*. *tetraquetra* [38]) is extinct, but available data indicate its mixing lexical composition with several Ainu dialects (including Sakhalin), and also a closer relationship with some Hokkaido dialects [e.g., 68]. All these data provide a strong evidence that while Ainu people reported to Steller the name *tochui*/*totsui*/*togoi* they mentioned an edible ascidian (most likely, *H*. *aurantium*), and not inedible nudibranch *T*. *tetraquetra*. We therefore disprove the common indication [22, 23, 42] about “edible *Tritonia tetraquetra*”. As a taxonomically important implication, the name *tochui* was incorrectly applied by Odhner ([23]: p. 50) to the species that currently known as *Tochuina gigantea*, and not *Tritonia tetraquetra* in the original sense of Pallas. All above information confirms that tritoniid species found by Steller on the Kuril Islands is *Tritonia*, but not a *Tochuina*.

### Consolidated data on phylogeny and taxonomy of the family Tritoniidae

Currently the classification of the family Tritoniidae is ambiguous and a modern taxonomic synopsis integrating morphological and molecular data is lacking. Odhner [23] and Marcus [69] recognized several genera and subgenera among tritoniid taxa. Thompson and Brown [1], Gosliner and Ghiselin [70] nevertheless used single genus *Tritonia*. However, some genera, e.g. *Tritoniopsis* Eliot, 1905 is continued to use currently. Instead, other taxa, e.g. *Tochuina* are sometimes omitted [19] in spite of the current valid status [71]. The genus *Marionia* Vayssière, 1877 is different from other tritoniid taxa because of presence of stomach plates, but otherwise contains numerous species with disparate morphology, and molecular phylogenetic data show within *Marionia* at least two distinct clades [5, 72]. The taxonomy of the type tritoniid genus *Tritonia* has been especially controversial. Several disparate morphological groups supported by molecular data currently falls within single genus *Tritonia*. Thus, an assessment of the taxonomic placement of the model species of the North Pacific tritoniids is impossible without a broad-scope phylogenetic framework for the family Tritoniidae. Otherwise it is not possible clearly indicate for what reason the model species *Tritonia tetraquetra* was assigned to the genus *Tochuina* for a long time and why *Tochuina* has some intriguing external and internal similarities to the non-tritoniid species *Doridoxa* Bergh, 1899 and *Heterodoris* Verrill et Emerton, 1882 (Figs 1A, 1B, 3 and 7). Furthermore, a morphologically highly aberrant genus *Marianina* (having the bifid cerata-like processesses instead of typically branched tritoniid dorsolateral appendages) is a sister to *Tritoniopsis*. The genus *Tritoniopsis* in turn is morphologically more similar to the type species of the genus *Tritonia* but phylogenetically is more close to the genera *Marianina*, *Tochuina* and *Tritoniella* (Fig 1A and 1B). Thus, unbalanced and a non-integrative classification regarding morphological and molecular data on various tritoniid lineages will emerge if the all tritoniid diversity will be united into a single genus *Tritonia*. Therefore, herein the taxonomic synopsis of the family Tritoniidae is presented and accommodates modern morphological and molecular discrepancies.

Previously, several working rules for integration of morphological and molecular data to produce a consistent classification were outlined [73]. One of the main principle, it is avoidance of large taxa containing many species. Another important rule, it is separation of a morphologically aberrant taxon, when it phylogenetically nested within taxa with disparate morphology. The present phylogeny of the family Tritoniidae is highly relevant case for application of such proposals. If a broad-taxa approach was applied, an inevitable synonymization of the almost all tritoniid diversity (including taxa *Tochuina* and *Marianina*) into a single genus must be used. The argumentation on the “presence of intermediate forms” [70] is not a relevant one because evolution does not proceed with an “intermediate” forms in its straightforward understanding. Phylogeny shows a complicated mosaic of various characters at many levels of phylogenetic differentiation in an ontogenetic framework. The evolutionary key linking taxa can be found, e.g. *Onchimira* that links cryptobranch and phanerobranch dorid nudibranchs [74], but this is not a reason for synonymyzation of morphologically highly disparate taxa. A potential lumping decision will mask taxonomic diversity and can be very impractical for potential descriptions on any further new species. The strict apomorphy-based diagnoses are also problematic. Any apparent apomorphy (as in case of tritoniids, a broad body or tricuspid central teeth) can change or disappear in a crown group due to the ontogenetic process of paedomorphosis [24]. For example it can be reliably applied to the sister tritoniid genera *Marianina* and *Tritoniopsis* demonstrating the profoundly different morphology at adults stages (Fig 1A and 1B). The application of narrow-defined genera makes focus on hidden diversity among particular, smaller lineages. Recognition of the monotypic genera is also in line with the consistent morphological and molecular units. This proposal is a practical one, because even supporters of a lumping classification now separate a morphologically disparate monotypic nudibranch genus *Bonisa* from a large paraphyletic assemblage of the genus *Janolus* [75].

**Fig 8:**
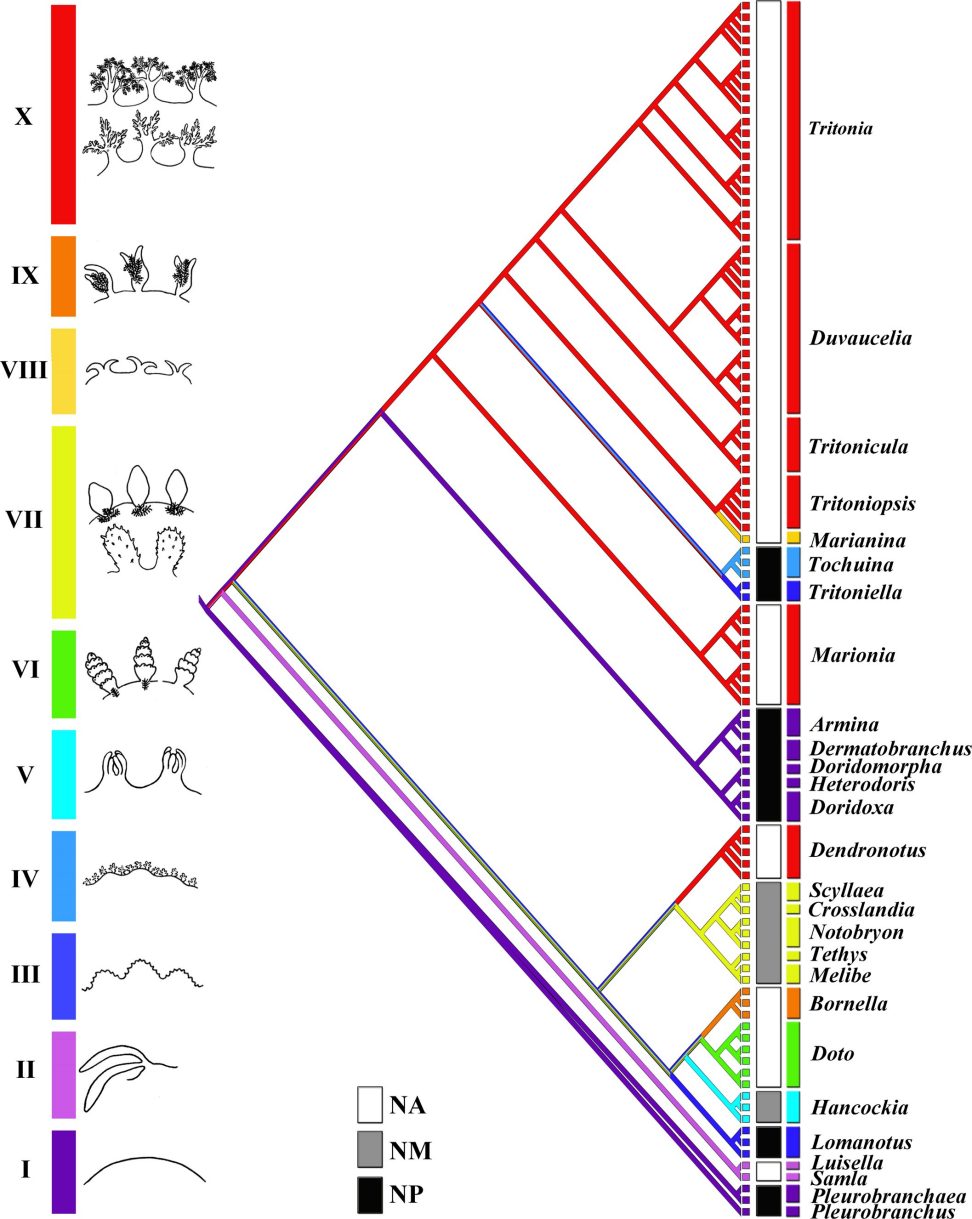
Ancestral character state reconstruction for the dorsolateral appendages/secondary gills and notum traits based on COI + 16S + H3 concatenated dataset inferred by Bayesian inference (BI). The following patterns of the dorsolateral appendages/secondary gills characters are recognized and colour-coded: I. Dorsolateral appendages absent or indistinct, cnidosacs absent. II. Dorsolateral appendages represent by non-branched, elongate cerata with single cnidosac per ceras. III. Dorsolateral appendages non-branched or weakly branched, numerous, from very short and indistinct to more elongate and distinct, commonly associated with notum and form scalloped lobes, cnidosacs absent. IV. Dorsolateral appendages branched, indistinct, numerous, cnidosacs absent. V. Dorsolateral appendages non-branched or partially branched, elongate and folded, distinct, few or moderate in number, with multiple cnidosacs per appendage, integrated to modified notal edges. VI. Dorsolateral appendages non-branched, cone-shaped with tubercles or folds, few or moderate in number, often accompanied by branched “gill-like” structures at base, cnidosacs absent. VII. Dorsolateral appendages represent by few large lobes (modified notal edges), often accompanied by branched “gill-like” structures covered these lobes or its bases, cnidosacs absent. VIII. Dorsolateral appendages bifid, distinct, few in number, cnidosacs absent. IX. Dorsolateral appendages branched, cerata-shaped, distinct, few in number, accompanied by branched “gill-like” structures, cnidosacs absent. X. Dorsolateral appendages branched, distinct, few or moderate in number, cnidosacs absent. For the notum following states are recognized (the details of codes on the figure): NA, notal edges are absent or weakly defined; NM, notal edges considerably modified (for example, into large separate lobes); NP, notal edges present, well-defined. Sources of the morphological data used for the character state coding are [1, 2, 25, 47, 73, 81, 86, 90, 93, 117, 127] and the present study. See also text for discussion.

Small genera and families are the reliable way to propose maximally coherent taxonomic units to accomodate morphological and molecular data [73, 76]. The long term confusion between two large common North Pacific tritoniid taxa *Tritonia tetraquetra* (previously known as “*Tritonia diomedea”*) and *Tochuina gigantea* (previously known as “*Tochuina tetraquetra*”) well illustrates the need to use this approach. The misidentification between these two distantly related tritoniid clades, *Tochuina* and *Tritonia* has persisted for more than a century. In the present study using combination of morphological data from the type specimen and recent molecular data we finally evidently demonstrate the significant differences between *Tochuina* and *Tritonia* in a broad phylogenetic framework (Figs 1–6). Compare to the genus *Tritonia*, genus *Tochuina* has distinctive features as presence of numerous, small, branched dorsolateral appendages, oral veil without elongate processes, and unicuspid central teeth (see Figs 2–4). Other genera of tritoniids, e.g. *Tritoniopsis* and *Tritoniella* may also possess unicuspid central teeth, but they differ from *Tochuina* by combination of other features. These characters include presence of fewer large branched appendages in *Tritoniopsis* and absence of distinctly branched dorsolateral appendages in *Tritoniella*. Such diagnoses are more complex than traditional ones (based on a searching for strict differences), but much finer encompasses the molecular and morphological data. Also, the genus *Tochuina* shows external similarity to the distantly related non-tritoniids *Doridoxa* and *Heterodoris* (compare Figs 3H–3L, 7A, 7D, 7E, 7K–7M and 7P–7S). A well recognized common species of the North Pacific tritoniids, *Tritonia festiva* (Stearns, 1873), though largely was not involved the neurobiological works, but closely related to the important model species, *T*. *tetraquetra* and *T*. *exsulans* (Figs 1A and 6). These similarities and differences need to be addressed. Family Tritoniidae needs a synopsis based on the available integrative data. Following valid genera (in alphabetical order) are proposed to recognize within the family Tritoniidae. The adult characters are listed in the diagnoses below because juvenile features can be different considerably. In addition in this study were specially investigated phylogenetic placement of Doridoxidae and Heterodorididae (Figs 1A, 1B and 8), and respective taxonomic diagnoses of these families are presented after synopsis of the family Tritoniidae.

### Taxonomic synopsis of the family Tritoniidae Lamarck, 1809

**Genus *Duvaucelia* Risso 1826, restricted**

= *Candiella* Gray, 1850

Type species. *Duvaucelia gracilis* Risso, 1826 (= *Tritonia manicata* Deshayes, 1853)

Diagnosis. Body narrow. Dorsolateral appendages distinct, branched, relatively few in number. Notal edge indistinct. Anterior corners of notum absent. Rhinophoral sheaths closed, without lateral opening and appendage. Oral veil not bilobed with few long processes. Anal opening usually placed towards anterior part of lateral side. Jaws oval. Masticatory edge with conical elements. Central radular teeth tricuspid. Moderate number of rows of lateral teeth, about 20–30 per half row. Stomach plates absent. Seminal receptacle relatively large, oval with long stalk and without large bag-like base. Copulative organ conical, without distinct folds. Usually small forms with body length no more than ca. 30 mm. Sources of the morphological data used in the diagnosis are indicated in the Table 3.

**Table 3:** Diagnostic characters of the genera in the family Tritoniidae.

Genus	Body	Oral veil	Rhinophoral sheaths	Notal edge	Dorsolateral appendages	Lateral teeth of radula	Central teeth	Stomach plates	Copulative organ	References
***Duvaucelia* Risso 1826**	Narrow, commonly up to 30 mm in length	Oral veil not bilobed with few long processes	Closed, without appendage; Anterior corners of notum absent	Indistinct	Distinct, branched, relatively few in number	Moderate in number, about 20–30 per half row	Tricuspid	Absent	Conical, without distinct folds (here and below conical structures of various length are implied, including “flagelliform” ones)	[1, 69; 77, 90]
***Marianina* Pruvot-Fol, 1931**	Very narrow, commonly up to 15 mm in length	Oral veil not bilobed with pair of long oral-tentacle like processes and pair of shorter appendages	Closed, with distinct appendage; Anterior corners of notum in front of rhinophores absent	Indistinct	Distinct, bifid, cerata-like, few in number	Small in number, about 5–6 per half row	With strong triangular cusp and distinct lateral denticles	Absent	Elongated, with rounded tip, without distinct folds	[46, 84, 85]
***Marionia* Vayssière, 1877 (a heterogeneous assemblage)**	Broad to moderately narrow, up to 200 mm in length, but small forms also present	Oral veil bilobed or not, with numerous long processes	Closed, without appendage; Anterior corners of notum absent or indistinct	Notal edge commonly remains as narrow folds between of dorsolateral appendages	Distinct, branched, from few to numerous	From moderate to extremely large number (from 15 to more than 150 teeth) per half row in various species	Tricuspid	Present	Conical or bulbous, without distinct fold	[3, 72, 87, 88 89, 90]
***Paratritonia* Baba, 1949**	Narrow, up to 30 mm in length	Oral veil not bilobed with few long processes	Closed, without appendage; Anterior corners of notum absent	Indistinct	Distinct, branched, few in number	Numerous, 100–110 per half row	Unicuspid	Present	Unknown	[91]
***Tochuina* Odhner, 1963**	Broad, up to 300 mm in length	Oral veil not bilobed without processes	Partly open laterally, lateral appendage absent; Anterior corners of notum present	Distinct	Indistinct, branched, numerous	Numerous, up to 200 teeth per half row	Unicuspid	Absent	Conical, without distinct folds	[17, 19, 23, 47, 48, 69; present study]
***Tritonia* Cuvier, 1798**	Broad, up to 300 mm in length	Oral veil bilobed with numerous long processes	Closed, without lateral opening and distinct appendage; Anterior corners of notum absent or indistinct	Indistinct	Distinct, branched, moderate to relatively few in number	Numerous, up to 200 teeth per half row	Tricuspid	Absent	Massive, cylindrical or conical, with variously expressed folds	[1, 23, 46, 47, 50, 53, 69, 93–98, 106; present study]
***Tritonicula* gen. nov.**	Narrow, commonly up to 20 mm in length	Oral veil not bilobed with few long processes	Closed, with elongate lateral appendage in some species; Anterior corners of notum absent	Indistinct	Distinct, branched, few in number	Small in number, up to 11 per half row	Tricuspid	Absent	Conical or rounded, without distinct folds	[14, 70, 78, 99]
***Tritonidoxa* Bergh, 1907**	Narrow, up to 30 mm in length	Oral veil not bilobed with few processes.	Closed, without lateral appendage; Anterior corners of notum indistinct	Distinct	Absent	Numerous, up to 75 per half row	Tricuspid	Absent	Conical, without distinct folds	[100]
***Tritoniella* Eliot, 1907**	Narrow to moderate, up to 80 mm in length	Oral veil not bilobed or indistinctly bilobed, without processes	Closed, with lateral expansion; Anterior corners of notum indistinct	Distinct	Absent or indistinct, non-branched	Numerous, more than 50 per half row	Unicuspid or tricuspid	Absent	Conical, with distinct circular fold or bulge	[102]
***Tritoniopsis* Eliot, 1905**	Moderate, up to 50 mm in length	Oral veil not bilobed with numerous long processes	Closed, sometimes with lateral appendage; Anterior corners of notum absent	Indistinct	Distinct, commonly very large, branched, relatively few to moderate in number	Moderate to numerous in number, about 30–50 per half row	With prominent triangular cusp and fine lateral denticles, or smooth	Absent	Conical, without distinct folds	[69, 103, 104]

Species composition. *Duvaucelia lineata* (Alder et Hancock, 1848), *Duvaucelia manicata* (Deshayes, 1853), *Duvaucelia nilsodhneri* (Marcus Ev., 1983) comb. nov., *Duvaucelia plebeia* (G. Johnston, 1828), *Duvaucelia striata* (Haefelfinger, 1963),? *Duvaucelia taliartensis* (Ortea et Moro, 2009) comb. nov.

Remarks. This is a maximal supported clade in the present phylogenetic analysis (Fig 1A) that constitutes a narrowly-defined group of a few species of tritoniids. This group also shows very consistent morphological features, such as relatively small size, not bilobed small oral veil and moderate number of lateral teeth. This narrowly-defined genus has support from biogeographical data since it encompasses species that inhabits mostly European temperate and subtropical waters. Therefore, the older genus *Duvaucelia* in the restricted sense is resurrected here (see list of included species above). For a species *Tritonia taliartensis* molecular or internal morphological data are not available from the original description [77], but general external appearance (small size, 7.5 mm and narrow body) similar to the species of the restricted genus *Duvaucelia*.

**Genus *Marianina* Pruvot-Fol, 1931**

= *Aranucus* Odhner, 1936

Type species. *Marianina rosea* (Pruvot-Fol, 1930) (= *Aranucus bifidus* Odhner, 1936)

Diagnosis. Body very narrow. Dorsolateral appendages distinct, bifid, cerata-like, few in number. Notal edge indistinct. Anterior corners of notum absent. Rhinophoral sheaths closed, without lateral opening and with distinct fleshy lateral appendage. Oral veil with two long processes pointed forward and two shorter processes directed laterally. Anal opening placed towards anterior part of lateral side. Jaws oval. Masticatory edge with small granule-like denticles. Central radular teeth with strong triangular cusp and distinct lateral denticles. Small number of rows of lateral teeth (about 5–6 per half row). Stomach plates absent. Seminal receptacle large, oval with short stalk and without large bag-like base. Copulative organ elongated, with a rounded tip, without distinct folds. Small forms with body length no more than 15 mm. Sources of the morphological data used in the diagnosis are indicated in the Table 3.

Species composition.? *Marianina khaleesi* (Silva, de Azevedo et Matthews-Cascon, 2014) comb.nov., *Marianina rosea* (Pruvot-Fol, 1930).

Remarks. The highly aberrant genus *Marianina* with bifid but not branched dorsolateral appendages is phylogenetically sister to the genus *Tritoniopsis* (Figs 1A and 8). The genus *Tritoniopsis* however has a “typical” tritoniid external morphology. This proves necessity of a fine-scale distinguishing of the genera within the family Tritoniidae. The radular patterns of adult *Marianina rosea* highly resemble early juvenile teeth of other complex at adult stage tritoniids [78, 79], and also species from the distantly related family Dendronotidae [80, 81]. According to the molecular phylogenetic analysis (Fig 1A) *Marianina* with simple external morphology (narrow body with non-branched appendages, Figs 1A, 1B and 8) is a sister taxon to the tritoniid *Tritoniopsis* with a complex external morphology (broad body with strongly branched appendages). The juvenile appearance of some set of characters and simultaneous phylogenetic placement inside of a group with generally complex morphology is a robust evidence for the paedomorphosis [24]. The disparate morphology as in *Marianina* and phylogenetic relation to a complex group is characteristic for paedomorphic taxa and commonly resulting in proposals for higher taxonomic groupings. Two genera were proposed two times for the same paedomorphic tritoniid species: *Marianina* Pruvot-Fol, 1930 [82] and *Aranucus* Odhner, 1936 [46]. The separate family Aranucidae was later synonymyzed with Marianinidae (now considered as a synonym of Tritoniidae). The process of paedomorphosis likely dominated in the evolution of the genus *Marianina*. Intriguingly, there is a species, currently assigned to the genus *Tritonia*, *T*. *khaleesi* Silva, Azevedo et Matthews-Cascon, 2014 which shows a remarkable combination of small size (up to 12 mm), a gracile external shape with just two long and two short processes of the oral veil, the radula with central teeth bearing strong triangular cusp with lateral denticles and a few lateral teeth (max 5) ([83]: pp. 580–582). These characters are significantly similar to these in *Marianina rosea* [46, 84, 85]. Reproductive systems are quite similar in both taxa, with distinct prostate, relatively large receptaculum seminis on a short stalk and copulative organ without distinct folds [46, 83]. “*Tritonia*” *khaleesi* demonstrates a very similar to *Marianina* strongly paedomorphic radular morphology. The only difference that *T*. *khaleesi* shows partly branched dorsolateral appendages, but some appendages are already unbranched ([83]: pp. 580–581). *Marianina rosea* and *T*. *khaleesi* also share similar fleshy lobe at the lateral sides of rhinophoral sheaths. A small tritoniid “*Tritonia*”*pickensi* (Marcus and Marcus, 1967) also shows partly similar to *Marianina rosea* and “*T*.” *khaleesi* few elongate processes of oral veil and a lateral lobe at rhinophoral sheaths. However radula of “*Tritonia*”*pickensi* has common for tritoniids tricuspid central teeth, whereas central teeth of “*T*.” *khaleesi* is very similar to *Marianina*. While there is a possibility that *T*. *khaleesi* is independently from *Marianina* acquired paedomorphic radular morphology, taking into consideration several significant similarities in different characters, it is more probable that *T*. *khaleesi* is more closely related to *M*. *rosea*. We therefore transferred here “*T*.” *khaleesi* into the genus *Marianina* (*Marianina khaleesi* (Silva et al., 2014) comb. nov.) with a reservation. When the molecular data for *M*. *khaleesi* will be available, it will be possible to further conclude either it is a sister species to *M*. *rosea*, or a separate new genus within some other tritoniid lineage. In any scenario, compare to *M*. *rosea*, *M*. *khaleesi* demonstrates remnants of the branching pattern of the dorsolateral appendages, which is common to the majority of tritoniids. This is very consistent with the results of the molecular analysis shows that simplified paedomorphic *Marianina* is sister to the externally complex *Tritoniopsis* (Fig 1A and 1B), which already acquired the central teeth with a strong pointed cusp.

**Genus *Marionia* Vayssière, 1877**

Type species. *Tritonia blainvillea* Risso, 1818 (= *Marionia berghii* Vayssière, 1879)

Diagnosis. Body broad to moderate. Dorsolateral appendages distinct, branched, from few to numerous in number. Notal edge commonly remains as narrow folds between of dorsolateral appendages. Anterior corners of notum absent or indistinct. Rhinophoral sheaths closed, without lateral opening and appendage. Oral veil bilobed or not, with numerous long processes. Anal opening usually placed towards middle or posterior part of lateral side. Jaws oval. Masticatory edge with elements of various degree and numbers. Central radular teeth tricuspid. From 15 to more than 150 lateral teeth per half row in various species. Stomach plates present. Seminal receptacle very large with long stalk and without bag-like large base. Copulative organ conical or bulbous, without distinct folds. Usually large forms with body length more than 30 mm (up to 200 mm), but several species apparently are small-sized. Sources of the morphological data used in the diagnosis are indicated in the Table 3.

Species composition. *M*. *abrahamorum* Silva, Herrero-Barrencua, Pola et Cervera, 2019, *M*. *albotuberculata* (Eliot, 1904), *M*. *arborescens* Bergh, 1890, *M*. *babai* Odhner, 1936, *M*. *bathycarolinensis* Smith et Gosliner, 2005, *M*. *blainvillea* (Risso, 1818), *M*. *cabindae* White, 1955, *M*. *chloanthes* Bergh, 1902, *M*. *cucullata* (Couthouy, 1852), *M*. *cyanobranchiata* (Ruppell et Leuckart, 1828), *M*. *dakini* (O'Donoghue, 1924), *M*. *distincta* (Bergh, 1905), *M*. *echinomuriceae* Jensen, 1994, *M*. *elongoviridis* Smith et Gosliner, 2007, *M*. *elongoreticulata* Smith et Gosliner, 2007, *M*. *fulvicola* Avila, Kelman, Kashman, et Benayahu, 1999, *M*. *gemmii* Almón, Pérez et Caballer, 2018, *M*. *ghanensis* Edmunds et Carmona, 2017, *M*. *hawaiiensis* (Pease, 1860), *M*. *kinoi* Angulo-Campillo et Bertsch, 2013, *M*. *levis* Eliot, 1904, *M*. *limceana* Silva, de Meirelles et Matthews-Cascon, 2013, *M*. *olivacea* Baba, 1937, *M*. *ramosa* Eliot 1904, *M*. *rubra* (Rüppell et Leuckart, 1828), *M*. *pellucida* Eliot, 1904, *M*. *platyctenea* (Willan, 1988), *M*. *pusa* Er. Marcus et Ev. Marcus, 1968, *M*. *pustulosa* Odhner, 1936, *M*. *semperi* Jensen, 1994, *Marionia tedi* Ev. Marcus, 1983, *M*. *vanira* Ev. Marcus et Er. Marcus, 1966, *M*. *viridescens* Eliot 1904.

Remarks. The genus *Marionia* in spite of the external similarity to the genus *Tritonia* possesses such a very distinctive feature as the solid chitinous stomach plates. However this large genus currently is united only by this single character. Intriguingly, stomach plates which are very similar to *Marionia* also present in a distantly related dendronotoidean family Tethydidae [86] (Figs 1A and 8). This again raises a question that taxonomy should be based not only on the drastically different characters, but on a fine-scale diagnostics. In this respect, an extremely broad range of other characters, including number of dorsolateral appendages (ranged from 7 to 100 pairs) and lateral teeth (ranged from 15 to 150 teeth) were mentioned for the genus *Marionia* [3, 71, 87–90]. This diversity exceeds ranges of character “variations” (at adult stage) in other tritoniid genera, and clearly indicates that several genus-level lineages are hidden within putatively the same “*Marionia*” genus. The available names such as *Marioniopsis* and *Paratritonia*, after clarification to which clades they actually belong can be used for further genus-level delineation among “*Marionia*”. For example, the so far monotypic genus *Paratritonia* possesses small-sized species with few dorsolateral appendages but with a large number of the radular lateral rows ([91]: pp. 84–86). Some other small-sized species of “*Marionia*” can be included into that genus, when molecular data will became available for the type genus *Paratritonia lutea* Baba, 1949. In case they will be inconsistent with *P*. *lutea* (for this species the unicuspid central teeth was reported [91]), new genera will be needed to separate for such forms.

Importantly, according to all available data *Marionia* invariably forms a separate clade from other tritoniids [5, 19, 72, 92; present study, Fig 1A]. Therefore, potentially *Marionia* with related genera *Marioniopsis* and *Paratritonia* (and more genera which have to be separated) should be placed in a separate family. In this connection, there is another considerable issue with the genus *Marionia* that affects all tritoniids. The majority of the available phylogenetic analyses [5, 72, 92] shows that *Marionia* renders the family Tritoniidae paraphyletic regarding Dendronotoidea. A revision of the large *Marionia* clade is out of scope of the present paper and pending a separate study.

**Genus *Paratritonia* Baba, 1949**

Type species. *Paratritonia lutea* Baba, 1949.

Diagnosis. Body narrow. Dorsolateral appendages distinct, branched, few in number. Notal edge indistinct. Anterior corners of notum absent. Rhinophoral sheaths closed, without lateral opening and appendage. Oral veil not distinctly bilobed with few long processes. Anal opening usually placed towards middle or posterior part of lateral side. Jaws oval. Masticatory edge with teeth-like elements of various degree and numbers. Central radular teeth unicuspid with additional denticles. Numerous rows of lateral teeth (100–110 per half row). Stomach plates present. Anterior and posterior portions of digestive gland fused. Reproductive organs unknown. Small forms with body length up to 30 mm. Source of the morphological data used in the diagnosis are indicated in the Table 3.

Species composition. Only type species so far was included [91].

Remarks. See under genus *Marionia*.

**Genus *Tochuina* Odhner, 1963**

Type species. *Tritonia gigantea* Bergh, 1904 (= “*Tritonia tetraquetra*” sensu Bergh, 1879 non Pallas, 1788)

Diagnosis. Body broad. Dorsolateral appendages indistinct, branched, numerous. Notal edge distinct, well-defined. Anterior corners of notum present, distinct. Rhinophoral sheaths partly open laterally; lateral appendage absent. Oral veil not bilobed without processes or with small tubercles. Anal opening placed towards middle or posterior part of lateral side. Jaws square. Masticatory edge smooth or possibly with indistinct elements. Central radular teeth unicuspid. Additional fine denticles often present on central teeth. Numerous rows of lateral teeth, more than 50 (up to 200) per half row. Stomach plates absent. Seminal receptacle relatively large with long stalk and without bag-like base. Copulative organ conical, without distinct folds. Large forms with body length more than 30 mm and include very large form up to 300 mm. Sources of the morphological data used in the diagnosis are indicated in the Table 3.

Species composition. *Tochuina gigantea* (Bergh, 1904), *Tochuina nigritigris* (Valdés, Lundsten et Wilson, 2018) comb. nov., *Tochuina nigromaculata* (Roginskaya, 1984) comb. nov.

Remarks. The incompletely closed rhinophoral sheaths and the well defined anterior notal corners of *T*. *gigantea* (Figs 3I and 3J) represent considerable similarity with the non-tritoniid taxa *Doridoxa walteri* (Krause, 1892) [93] (Figs 7A, 7D, 7E and 7K–7M) and *Heterodoris robusta* Verrill et Emerton 1882 (Fig 7P–7S), see also Discussion). In the present study we for the first time perform the molecular analysis with inclusion of the type species of the genus *Tochuina*, *T*. *gigantea* (previously incorrectly identify as “*Tochuina tetraquetra*”). As results, we robustly reveal that a recently described species which was assigned to “*Tritonia*”, *T*. *nigritigris* from NE Pacific [19] forms instead a maximal supported clade with the type species of the genus *Tochuina* (Fig 1A). The species *T*. *nigritigris* is therefore transferred to the genus *Tochuina*. Valdés et al. [19] while described *T*. *nigritigris* did not mention that this species is very similar (including name) to the species that was described considerably earlier by Roginskaya in 1984 [94] as *Tritonia nigromaculata* also from the deep sea of the North Pacific (Fig 3S–3Z). Here we present the first SEM images of the radula of a paratype of *“Tritonia” nigromaculata* (Fig 3W–3Z). The central teeth are unicuspid with additional denticles, the cusp is massive and long (Fig 3Y). These patterns are very similar to the original descriptions of *T*. *nigromaculata* [94] and *T*. *nigritigris* [19]. It should be specially noted, that the present detailed SEM study (Fig 3Y1 and 3Y2) did not reveal any peculiar “articulated” appendage of the central cusp (depicted in the original description of *T*. *nigromaculata* [94]: fig b on the p. 100). Also, the cusp is smooth (both in the anterior/middle and posterior parts of the radula, Fig 3W, 3X, 3Y1 and 3Y2), whereas originally the cusp was described as a “denticulated spine” [94: p. 101]. In addition, the thinner and delicate apical part of the cusp appears somewhat bent in the posteriormost teeth (Fig 3Y2) after standard processing of the radula in sodium hypochlorite solution. Nonetheless, the posteriormost teeth also do not possess an “articulated” cusp (Fig 3Y2). These differences are clearly due to a misinterpretation of the light microscopic data in the original description of *T*. *nigromaculata* [94]. This is significant because the incorrect data on the morphology of the cusp were used to consider the central teeth of *T*. *nigromaculata* as a unique structure of a “neotenous” origin [94: p. 104]. In reality, similar unicuspid teeth with a long, smooth cusp (partially denticulated mostly at the base, and without any “articulated spines”) are present in the different non-tritoniid taxa *Doridoxa* and *Heterodoris* (Fig 7H–7J, 7W and 7X), and the type species of the tritoniid genus *Tochuina* (Fig 3Q). The similarity of the unicuspid central teeth between “*Tritonia” nigromaculata* and *Tochuina gigantea* was previously underestimated [94]. The paedomorphosis is important evolutionary force [24] and attested in this study for several tritoniids. However, the case of *T*. *nigromaculata* suggests not only a paedomorphic trait in a particular *Tochuina* species, but rather a common phylogenetic pattern between Tritoniidae, Doridoxidae and Heterodorididae (see details below).

“*Tritonia*” *nigritigris* was indicated [19: p. 407] as “the most similar species…” with a species *Tritonia newfoundlandica*, which however is highly inconsistent with *T*. *nigritigris* because of the substantial differences in the oral veil, dorsolateral appendages and radula. Instead, both *T*. *nigritigris* and *T*. *nigromaculata* share similar non-bilobed oral veil without long processes, numerous small dorsolateral appendages, and unicispid central teeth. “*Tritonia*” *nigritigris* and *T*. *nigromaculata* also inhabit similar depths around 2000 m in the respective locations of the north-eastern and north-western Pacific, and potentially may represent the same species. In the present study we therefore correct the assessment in [19], and include “*Tritonia” nigromaculata* into the genus *Tochuina*. Morphologically, both *T*. *nigritigris* and *T*. *nigromaculata* share with the type species of the genus *Tochuina* (Fig 3A–3R) numerous small dorsolateral appendages, absence of frontal veil appendages and unicuspid central tooth (Fig 3S–3Z). The usual for *Tochuina gigantea* partially opened rhinophoral sheaths, instead are less pronounced in *T*. *nigromaculata* and *T*. *nigritigris*. “*Tritoniopsis*” *aurantia* Mattox, 1955 was considered as a synonym of *T*. *gigantea* [69], that needs to be checked with additional data.

**Genus *Tritonia* Cuvier, 1798, restricted**

= *Sphaerostoma* MacGillivray, 1843

= *Lateribranchiaea* Stearns, 1873

= *Candellista* Iredale et O'Donoghue, 1923

Type species. *Tritonia hombergii* Cuvier, 1803

Diagnosis. Body broad to moderate. Dorsolateral appendages distinct, branched, relatively few to moderate in number. Notal edge commonly indistinct. Anterior corners of notum absent or indistinct. Rhinophoral sheaths closed, without lateral opening and distinct appendage. Oral veil bilobed with numerous long processes. Anal opening usually placed towards middle or posterior part of lateral side. Jaws oval. Masticatory edge with strong conical or elongate bristle-like elements. Central radular teeth tricuspid. Additional fine denticles may present irregularly on central teeth. Usually many rows of lateral teeth, more than 50 (up to 200) per half row. Stomach plates absent. Seminal receptacle small or larger, oval with long stalk and a bag-like large base. Copulative organ massive, cylindrical or conical, with variously expressed folds. Usually large to moderate in size forms with body length more than 30 mm (including very large species up to 300 mm). Sources of the morphological data used in the diagnosis are indicated in the Table 3.

Species composition. *Tritonia antarctica* Pfeffer in Martens et Pfeffer, 1886, *T*. *australis* (Bergh, 1898), *T*. *bollandi* Smith et Gosliner, 2003, *T*. *challengeriana* Bergh, 1884,? *T*. *coralliumrubri* Doneddu, Sacco et Trainito, 2014, *T*. *dantarti* Ballesteros et Avila, 2006, *T*. *episcopalis* Bouchet, 1977, *T*. *exsulans* Bergh, 1894, *T*. *festiva* (Stearns, 1873), *T*. *griegi* Odhner, 1922, *T*. *hombergii* Cuvier, 1803, *T*. *incerta* Bergh, 1904, *T*. *indecora* Bergh, 1907, *T*. *ingolfiana* (Bergh, 1899), *T*. *newfoundlandica* Valdés, Murillo, McCarthy et Yedinak, 2017, *T*. *odhneri* Er. Marcus, 1959, *T*. *olivacea* Bergh, 1905, *T*. *pallescens* Eliot, 1906, *T*. *pallida* Stimpson, 1855, *T*. *primorjensis* Minichev, 1971, *T*. *tetraquetra* (Pallas, 1788), *T*. *psoloides* Aurivillius, 1887, *T*. *vorax* (Odhner, 1926).

Remarks. Based on the present molecular phylogenetic analysis (Fig 1A) we restrict the genus *Tritonia* to several predominantly large forms with a numerous processes on the bilobed oral veil and a large number of the lateral teeth. In our present analysis this morphological group forms a clade that closely related to the type species *T*. *hombergii* and including these model species *T*. *tetraquetra* and *T*. *exsulans* (Figs 1–6). The genus *Tritonia*, even in the restricted form, still represents a partly heterogeneous taxonomic assemblage that pending further revision. In addition, the supports of molecular clades inside of the apparent “genus” *Tritonia* are not always high (Fig 1A). More lineages are deserved separate genus status. For example a species currently assigned to *Tritonia*, *T*. *flemingi* (Powell, 1937) is inconsistent with the morphological data on the type and closely related to it *Tritonia* species. Particularly, “*T*.*” flemingi* is a very small species, with non-bilobed oral veil with only few processes, and indistinct dorsolateral appendages [95, 96]. By these characters, “*T*.*” flemingi* partly approaches the genera *Duvaucelia* and *Tritonicula* gen. nov., but because of significant geographic gap it may represents an independent lineage of small-sized tritoniids. Therefore “*T*.*” flemingi* does not include here into the genus *Tritonia* pending molecular phylogenetic data to propose a separate genus for this species. For several other species, including for instance a deep-sea *T*. *griegi* Odhner, 1922, the molecular data are also still unavailable, and inclusion of these taxa into the genus *Tritonia* needs to be confirmed. Also not all included species are consistent morphologically with the type species *T*. *hombergi* from the northern Atlantic and related North Pacific *T*. *tetraquetra* and *T*. *exsulans*. For instance, Valdés et al. [19] compared two recently described species, *T*. *newfoundlandica* with *T*. *nigritigris*. However, according to the present analysis the deep-sea species from NE Pacific *T*. *nigritigris* belongs to the genus *Tochuina*, which is distantly related to the genus *Tritonia* (Fig 1A). The North Atlantic *T*. *newfoundlandica* instead shows presence of the bilobed veil with distinct processes and tricuspid central teeth [97], which are common in the genus *Tritonia*, but not in *Tochuina*. We therefore keep *T*. *newfoundlandica* in the genus *Tritonia*. Because of several genus-level synonyms of the genus *Tritonia* are available (see synonymy above), some of them will be needed to resurrect in a future. In addition, the present *Tritonia* species composition is inconsistent biogeographically. While the type species *T*. *hombergii* from the northern Atlantic, and the North Pacific species such as *T*. *tetraquetra*, *T*. *exsulans* and *T*. *festiva* are closely related with a maximal support (Fig 1A), another lineage of the Antarctic and Subantarctic species *Tritonia cf*. *antarctica* and *T*. *challengeriana* forms a basal clade with lower support to other *Tritonia* species (Fig 1A). *Tritonia cf*. *antarctica* and *T*. *challengeriana* previously were synonymyzed (a taxonomic review in [98]). However present analysis shows them as two distinct species (Fig 1A). According to the present molecular phylogeny, the Antarctic and Subantarctic tritoniid species likely represent a different genus, that needs to be addressed in a separate study. *Myrella* Odhner, 1963 (= *Microlophus* Mabille et Rochebrune, 1889, non *Microlophus* Duméril et Bibron, 1837) [23] is an available potential genus name for the clade of the Antarctic and Subantarctic tritoniids.

***Genus Tritonicula* gen. nov.**

Urn:lsid:zoobank.org:act: 7E6448CD-78DA-4A65-8141-0A9EB40D0D0C.

Type species. *Tritonia hamnerorum* Gosliner et Ghiselin, 1987

Etymology. From common family stem *Tritoni-* and a diminutive Latin suffix–*cula* in reference to a “little *Tritonia*” because the new genus contains so far only small, gracile species.

Diagnosis. Body usually narrow. Dorsolateral appendages distinct, branched, relatively few in number. Notal edge commonly indistinct. Anterior corners of notum absent. Rhinophoral sheaths closed, without lateral opening, in some species with elongate lateral appendage. Oral veil not bilobed with few long processes. Anal opening usually placed towards middle lateral side, or partly moves to dorsal side. Jaws oval. Masticatory edge with delicate tubercle-like elements or more distinct conical plates. Central radular teeth tricuspid. Additional fine denticles may present irregularly on central teeth. Few rows of lateral teeth, so far reported no more than 11 per half row. Stomach plates absent. Seminal receptacle relatively large with long stalk and without large bag-like base. Copulative organ conical or rounded, without distinct folds. Small forms with body length no more than 20 mm. Sources of the morphological data used in the diagnosis are indicated in the Table 3.

Species composition. *Tritonicula bayeri* Ev. (Marcus et Er. Marcus, 1967) comb. nov., *Tritonicula hamnerorum* (Gosliner et Ghiselin, 1987) comb. nov., *Tritonicula myrakeenae* (Bertsch et Osuna, 1986) comb. nov., *Tritonicula pickensi* Ev. (Marcus et Er. Marcus, 1967) comb.nov.,? *Tritonicula wellsi* (Er. Marcus, 1961) comb. nov.

Remarks. In the present analysis we confirm a separate and morphologically distinct clade (Fig 1A and 1B) from both *Tritonia* s. str. and *Duvaucelia* s.str. This group contains generally smaller-sized species (with body length no more than 20 mm) such as “*T”*. *bayeri* Ev. Marcus et Er. Marcus, 1967, “*T*.*” hamnerorum* Gosliner et Ghiselin, 1987, “*T*.*” myrakeenae* Bertsch et Osuna, 1986 and “*T*.*” pickensi* Ev. Marcus et Er. Marcus, 1967 [70, 78, 99]. All these species share small size, small number oral veil processes and a very small number of rows of lateral teeth (around 10 per half radular row) in adult specimens. This group of species is separated here as a new genus. This combination distinguishes *Tritonicula* gen. nov. from both generally large-bodied *Tritonia* and small-sized *Duvaucelia*. The genus *Tritonia* possesses in adult specimens bilobed oral veil with numerous processess and more than 50 lateral teeth per row. Small-sized *Duvaucelia* commonly has larger number of lateral teeth than *Tritonicula* gen. nov. (more than 20 lateral teeth per row), though in few species this number can be smaller. Also species of the genus *Duvaucelia*, compare to *Tritonicula* gen. nov. does not have lateral extension on the rhinophoral sheaths. In addition, the new genus is consistent biogeographically. All included species inhabit warm waters of the American Atlantic and the Caribbean, or neighbour tropical Pacific waters. *Tritonia wellsi* was originally described from North Carolina (and further founded in Brazil) and placed in the subgenus *Tritonidoxa* [14]. However, the real genus *Tritonidoxa* demonstrates completely different characters including absence of dorsolateral appendages [100]. Small size, few oral veil processes and no more than 10 lateral teeth per row of “*T*.” *wellsi* are consistent with *Tritonicula* gen. nov. We include this species into new genus with reservation because of absence of molecular data. A species “*T*.” *khaleesi* (for which there are no available molecular data) which is included here to the genus *Marianina* (see above) can be also potentially related to *Tritonicula* gen. nov. According to our analysis it is likely that *Duvaucelia* and *Tritonicula* gen. nov. acquired small body size and smaller number of the lateral teeth independently (Fig 1A). This is an example of importance of fine-scale taxonomic delimitation not only at the species level (as in the case of the delimitation of the model species *T*. *tetraquetra* and *T*. *exsulans*), but also at the genus level, because otherwise such convergent evolutionary events will be considerably masked.

**Genus *Tritonidoxa* Bergh, 1907**

Type species. *Tritonidoxa capensis* Bergh, 1907

Diagnosis. Body narrow. Dorsolateral appendages absent. Notal edge distinct. Anterior corners of notum indistinct. Rhinophoral sheaths closed, without opening laterally and lateral appendage. Oral veil not bilobed with few processes. Anal opening placed towards posterior part of lateral side. Jaws oval. Masticatory edge with conical elements. Central radular teeth tricuspid. Large number of rows of lateral teeth (ca. 75 per half row). Stomach plates absent. Seminal receptacle relatively large. Copulative organ conical, without folds. Body length about 30 mm. Source of the morphological data used in the diagnosis are indicated in the Table 3.

Species composition. Only type species, *T*. *capensis* [100].

Remarks. There are more species that could potentially be included in *Tritonidoxa* [69, 78, 101]. However all they are inconsistent with characters of the type species, first of all absence of the dorsolateral appendages. Thus, only single type species is included here.

**Genus *Tritoniella* Eliot, 1907**

Type species. *Tritoniella sinuata* Eliot, 1907

Diagnosis. Body narrow to moderately broad. Dorsolateral appendages absent or indistinct, non-branched. Notal edge distinct. Anterior corners of notum indistinct. Rhinophoral sheaths closed, without lateral opening, but expanded laterally. Oral veil not bilobed or indistinctly bilobed, without processes. Anal opening placed towards middle part of lateral side. Jaws square. Masticatory edge with fine tubercles. Central radular teeth in adults unicuspid or tricuspid. Additional distinct cusps may present on central teeth. Numerous rows of lateral teeth, commonly more than 50 per half row. Stomach plates absent. Seminal receptacle relatively large with long stalk and without large bag-like base. Copulative organ conical, with distinct circular fold or bulge. Large forms up to 80 mm. Source of the morphological data used in the diagnosis are indicated in the Table 3.

Species composition. *Tritoniella belli* Eliot, 1907 (= *T*. *sinuata* Eliot, 1907).

Remarks. The Antarctic genus *Tritoniella* well distinguishes from the majority of tritoniids by absence of distinctly branched dorsolateral appendages. Phylogenetically it is related to the genus *Tochuina* and important for the present study. Here it is shown that the Antarctic *Tritoniella* forms a sister clade to the North Pacific genus *Tochuina* (Fig 1A), but considerably differs from the latter by external morphological features. Therefore, without broader framework it is impossible to assess taxonomic position of particular model species. Furthermore, all so far known species of the genus *Tochuina* always possess unicuspid central tooth (Fig 3), whereas *Tritoniella* may possesses both unicuspid and tricuspid central teeth [102]. These data clearly demonstrate that straightforward taxonomic diagnoses like a dilemma “presence/absence” of a character should be substituted with a fine-scale diagnostics. This case also help for taxonomic studies to become really integrative one and consistent with the novel molecular data.

**Genus *Tritoniopsis* Eliot, 1905**

= *Tritoniopsilla* Pruvot-Fol, 1933

Type species. *Tritoniopsis brucei* Eliot, 1905

Diagnosis. Body moderately broad. Dorsolateral appendages distinct, commonly very large, branched, relatively few to moderate in number. Notal edge indistinct. Anterior corners of notum absent. Rhinophoral sheaths closed, without lateral opening, sometimes with a small lateral appendage. Oral veil not distinctly bilobed with numerous long processes. Anal opening usually placed towards middle part of lateral sides. Jaws square. Masticatory edge with conical elements. Central radular teeth elongate, with prominent elongate cusp, smooth of with a number of small, regularly arranged lateral denticles. Number of rows of lateral teeth moderate to numerous (ca. 30–50 per half row). Stomach plates absent. Seminal receptacle relatively large, oval with long stalk and without large bag-like base. Copulative organ conical, without distinct folds. Small to medium forms with body length from ca. 20 to 50 mm. Sources of the morphological data used in the diagnosis are indicated in the Table 3.

Species composition. *T*. *brucei* Eliot, 1905, *T*. *cincta* (Pruvot-Fol, 1937), *T*. *elegans* (Audouin, 1826), *T*. *frydis* Er. Marcus et Ev. Marcus, 1970.

Remarks. Type species of the genus *Tritoniopsis*, *T*. *brucei* Eliot, 1905 comes from temperate southern Atlantic (Gough Island). Other currently assigned to the genus *Tritoniopsis* species come from tropical and subtropical waters. The molecular data are not available for *T*. *brucei* and phylogenetic placement of the genus *Tritoniopsis* is based on other included species (Fig 1A). *Tritoniopsis* characterizes by the central tooth with a prominent triangular cusp, often with lateral denticles [103, 104], which is distinctive from the common in Tritoniidae tricuspidate teeth. The genus *Tritoniopsis* is a good example of the necessity of a fine-scale assessment of the genera within the family Tritoniidae. According to the phylogenetic data *Tritoniopsis* is closely related to the profoundly aberrant, paedomorphic genus *Marianina* (present study, Fig 1A and 1B), but external morphology of *Tritoniopsis* is consistent with the majority of other Tritoniidae [104]. In addition to the taxonomic synopsis of the family Tritoniidae, diagnoses and remarks for the phylogenetically related but morphologically different non-tritoniid families Doridoxidae and Heterodorididae (adressed in the present study, Figs 1, 7 and 8) are provided below.

### Diagnoses of the families Doridoxidae and Heterodorididae

**Family Doridoxidae** Bergh, 1899

Diagnosis. Body moderately broad. Dorsolateral appendages absent. Notal edge distinct, well-defined. Anterior corners of notum present, distinct. Rhinophoral sheaths partly open laterally; lateral appendage absent. Rhinophores perfoliate. Oral veil not bilobed without processes. Anal opening placed towards posterior part of lateral side. Jaws square. Masticatory edge smooth or possibly with indistinct elements. Central radular teeth with distinct, broad, triangular cusp. Additional fine denticles may present on central teeth. Moderate number of lateral teeth, up to ca. 30 per half row. Stomach plates absent. Digestive gland without distinct external lobes. Seminal receptacle with long stalk and without bag-like base. Copulative organ conical, without distinct fold. Medium to small-sized forms with body length ca. 20–30 mm. The sources of the morphological data are in [93, 97, 105–109] and present study (Fig 7A–7O).

Composition. The genus *Doridoxa* Bergh, 1899 contains confirmed species *D*. *walteri* (Krause, 1892) (= *Doridoxa ingolfiana* Bergh, 1899, syn. nov., see details below) and a second poorly known South African species *D*. *benthalis* Barnard, 1963 [110].

Remarks. The genus *Doridoxa* and family Doridoxidae until recently was among the most enigmatic and poorly known nudibranch taxa (see details in the Discussion section). Molecular data obtained in the present study and available in GenBank clearly suggest that *Doridoxa ingolfiana* Bergh, 1899 [105, 106] is a junior synonym of *Doridoxa walteri* (Krause, 1892) [108] (= *D*. *ingolfiana* Bergh, 1899 syn. nov.). The external and internal morphology of the described from the Barents Sea *D*. *walteri* [108] (Fig 7A and 7D–7O) is essentially similar to the previously redescribed *D*. *ingolfiana* from the waters of the northern Atlantic [107, 108, Fig 7B and 7C]. In the original description of *D*. *ingolfiana* (including “*D*. *inglofiana* var.”) the presence of both smooth and denticulate central teeth is indicated [105, 106] (Fig 7B and 7C). Our conclusions based on *D*. *walteri* from the type locality region (Barents Sea off the East Spitzbergen) well agreed with the original description of *D*. *walteri* in Krause [109] (outlined on Fig 7A) and further records [93, 111]. Exactly as reported for *D*. *ingolfiana*, *D*. *walteri* has both smooth and denticulate central teeth (Fig 7H–7J, 7N and 7O). Maximal uncorrected COI p-distances between *D*. *walteri* and “*D*. *ingolfiana*” are 0.91% (Table 2), and according ABGD analysis they are the same species. The taxonomic placement of *Doridoxa benthalis* Barnard, 1963 [110] needs to be clarified since only single poorly preserved specimen is available [107]. *D*. *benthalis* does not demonstrate considerable similarity [110] to the type species of the genus *Doridoxa* and may represent a separate taxon of the genus or family levels.

**Family Heterodorididae** Verrill et Emerton, 1882

**(=** Atthilidae Bergh, 1899)

Diagnosis. Body moderately broad. Dorsolateral appendages non-branched or absent. Notal edge distinct, well-defined. Anterior corners of notum present, distinct. Rhinophoral sheaths partly open laterally; lateral appendage absent. Rhinophores perfoliate. Oral veil not bilobed without processes. Anal opening placed towards posterior part of lateral side. Jaws oval. Masticatory edge smooth or possibly with indistinct elements. Central radular teeth unicuspid with a narrow, weak cusp. Additional fine denticles may present on central teeth. Numerous lateral teeth, more than 50 (up to 200 and more) per half row. Stomach plates absent. Digestive gland with several external lobes. Seminal receptacle with long stalk and without bag-like base. Copulative organ conical or cylindrical, without distinct fold. Medium sized forms with body length up to ca. 35 mm. The sources of the morphological data are in [104, 107, 112–115] and present study (Fig 7P–7X).

Composition. The genus *Heterodoris* Verrill et Emerton, 1882 (= *Atthila* Bergh, 1899, see details below) contains two confirmed species: *H*. *robusta* Verrill et Emerton 1882 and *H*. *anthipodes* Willan, 1981.

Remarks. The genus *Heterodoris* and the family Heterodorididae show intriguing similarity to the family Doridoxidae (which was previously widely discussed as a key taxon for nudibranch phylogeny [107], Fig 7) and to the tritoniid genus *Tochuina* (Fig 3). However prior to this study (Fig 1A) the molecular data on Heterodorididae were never presented and incorporated into a molecular phylogeny. Present phylogenetic analysis shows complex pattern of the molecular relations and morphological disparities (Fig 1A and 1B). The family Heterodorididae is revealed as the sister group of the morphologically very different dorid-like Doridomorphidae (Fig 1A). The family Doridoxidae (morphologically similar to Heterodorididae), in its turn, comes as the sister group of Doridomorphidae+Heterodorididae clade (Fig 1A). Together Doridoxidae, Heterodorididae, and Doridomorphidae are sister to the morphologically highly disparate family Arminidae (Fig 1A). The family Arminidae differs from Heterodorididae and Doridoxidae substantially, by presence of joined rhinophores with vertical lamellae and oral veil with a strong transversal fold (Fig 1A and 1B, see also Discussion). Thus, in case of an artificial taxonomic unification of Heterodorididae with Arminidae or Doridoxidae, the synonymyzation of Arminidae and drastically different family Doridomorphidae will also be required. This will be a counterproductive decision, because instead of producing of a fine-scale taxonomic system, different taxa will be lumped into a large assemblage without support of apomorphies. Therefore, the present study confirms both *Doridoxa* and *Heterodoris* warrant separate families Doridoxidae Bergh, 1899 and Heterodorididae Verrill et Emerton 1882 (reinstated) respectively. The external similarity between the families Doridoxidae and Heterodorididae, as well as similarity to the tritoniid genus *Tochuina* are likely due to the plesiomorphic similarity of the rhinophoral sheaths and oral veil patterns (see details below). Internally families Doridoxidae and Heterodorididae are different in presence of an entire (except for microscopical branches [107]) digestive gland in Doridoxidae and subdivided into few lobes [113] in Heterodorididae. However, for the second species *H*. *anthipodes* the lobed digestive gland was not reported [114] and this character needs in a further investigation.

The genus *Atthila* Bergh, 1899 and the family Atthilidae, introduced by Bergh [106], are synonyms of *Heterodoris* Verrill et Emerton, 1882 and the family Heterodorididae Verrill et Emerton 1882 respectively. It was already proposed [113], but later on it was suggested to restore the genus *Atthila* without a study of additional materials [115]. However, both *Atthila* and *Heterodoris* show the same incompletely closed rhinophoral sheaths integrated to the oral veil [106, 113, present study]. This character was misleadingly described for *Atthila* as a “two-lobed edge of rhinophoral cavity” [106, 115]. *Atthila* and *Heterodoris* also come from the same geographic region of the western part of the North Atlantic and similar depth range (800–1500 meters) [106, 111, present study]. The conspecificity of deeper records of *H*. *robusta* from more southern localities in the north-eastern Atlantic [116] and exact number of species of the genus *Heterodoris* worldwide needs in a further investigation, including clarification of the species status “*Heterodoris ingolfiana*” [106, 116]. In this study several specimens of *Heterodoris robusta* from the type locality region of both *Heterodoris* and *Atthila* (the northern Atlantic coast of North America) were investigated. It was discovered that both denticulated and non-denticulated central teeth may occur in the same exemplar (Fig 7W and 7X). This is important result because prior to this study the denticluated versus non- denticulated central tooth was proposed as a main distinguishing character between *Atthila* and *Heterodoris* [115]. Also, denticulation on the central teeth of *H*. *robusta* represents by weak folds (Fig 7W and 7X) and could be omitted in course of a light microscopic study by Odhner [113]. Furthermore, variation of the body length [115] and internal characters (number and patterns of the lateral teeth) do not show any significant differences between *Heterodoris* and *Atthila*. Thus, there are no reliable characters to distinguish *Heterodoris* and *Atthila* and the latter genus is considered as synonym of *Heterodoris*.

### Phylogenetic relations of the family Tritoniidae to other nudibranch groups

The family Tritoniidae was recently shown as more closely related to the family Arminidae than to other traditional dendronotacean nudibranchs [4, 117]. Arminidae have no branched notal appendages and possess different rhinophores and anterior notum. The placement of the family Tritoniidae together with Arminidae renders traditional Dendronotacea paraphyletic. However, the taxon selection of the arminids and related families was limited and previously included only common shallow water arminid genera *Armina* and *Dermatobranchus*. The tritoniid genus *Marionia* and related taxa were also not included into that previous analysis [117]. In the present study the non-tritoniid genera *Doridoxa* and *Heterodoris* (Figs 1 and 7) from the deep sea of the North Atlantic were included into phylogeny of the family Tritoniidae for the first time. These non-tritoniid taxa are important for a tritoniid phylogeny consolidation. *Doridoxa* and *Heterodoris* have protruding anterior corners of the notum and rhinophoral sheaths with a lateral opening considerably similar to the tritoniid genus *Tochuina* (Figs 3 and 7). Arminidae have joined rhinophores without sheaths and usually with vertical lamellae (instead of horizontal ones in *Doridoxa* and *Heterodoris*) (Figs 1 and 7); these patterns are very different from Tritoniidae. By presence of perfoliated rhinophores both *Doridoxa* and *Heterodoris* are also different from the tritoniid *Tochuina*. However, the unicuspid central teeth of the radula in *Doridoxa* and *Heterodoris* is substantially similar to the tritoniid genera *Tochuina* and *Tritoniopsis* (Figs 3 and 7). This highlights the necessity to include *Doridoxa* and *Heterodoris* into molecular study of the family Tritoniidae. The molecular data on the North Atlantic *Heterodoris robusta* and for *Doridoxa walteri* from the Barents Sea are presented here for the first time (Figs 1 and 8).

According the molecular phylogenetic data, *Doridoxa*, *Heterodoris* and *Doridomorpha* clades are sister to the non-tritoniid family Arminidae (Fig 1). Similar externally *Tochuina* (Fig 3) comes as a basal lineage within the family Tritoniidae (Fig 1). Two phylogenetically distantly related lineages as Doridoxidae and Heterodorididae from one hand and tritoniid *Tochuina* on other hand have similar widely separated rhinophores with not completely closed rhinophoral sheaths integrated to the oral veil (Figs 1, 3 and 7). Instead, the peculiar joined rhinophores are restricted solely to the family Arminidae (Fig 1A and 1B). The transversal fold of the oral veil (Fig 1B) is also unique feature of the family Arminidae. Furthermore, among majority of the members of the distantly related superfamilies Tritonioidea and Dendronotoidea the separate non-joined rhinophores and oral veil without transversal fold are common character states (Fig 1A and 1B). The widespread presence of non-joined rhinophores supports separate rhinophores as plesiomorphic condition for Tritonioidea and Dendronotoidea in which lateral rhinophoral sheath opening was closed during subsequent phylogenetic diversification (Fig 1B). Available ontogenetic data for the type species of the genus *Tritonia* show earlier appearance of separate rhinophores integrated to the oral veil [79] and also support plesiomorphic state of this character. Taking together, the morphological and molecular phylogenetic data indicate separate rhinophores as a common ancestral character instead of a convergence. At the same time the structure of rhinophores in *Doridoxa* and *Heterodoris* (perfoliated, horizontal lamellae) differ from *Tochuina* (vertical, commonly branched lamellae typical of tritoniids). These taxa belong to different families, which acquired specialized characters in course of the phylogeny (Fig 1).

The majority of tritoniids do not have well-defined anterior corners of notum at adult stages (Fig 1A and 1B). However, early juveniles of *Tritonia* possess distinct notal anterior corners and rhinophores integrated to the oral veil [79]. During subsequent development of *Tritonia* towards adult stages, anterior corners of notum considerably reduced, whereas rhinophoral sheaths instead become distinct, but still integrated to the posterior part of the oral veil [79]. All members of Doridoxidae and Heterodorididae at the adult stages have well-defined anterior corners of notum integrated with the rhinophores and posterior part of the oral veil (Figs 1 and 7), thus similar in this respect to the early juveniles of *Tritonia*. This is a reliable ontogenetic evidence for the plesiomorphic state of the distinct anterior corners of notum and ancestral integration of the oral veil and rhinophores. Among tritoniids only few genera, e.g. *Tochuina* and *Tritoniella* possess distinct corners of the anterior notum at the adult stages (Fig 1A and 1B). The well-defined anterior corners of notum in combination with closed rhinophoral sheaths present also in the other families (e.g. Curnonidae from Antarctic and Lemindidae from South Africa), which are distantly related to the family Tritoniidae [115, 118–121]. These ontogenetic and molecular phylogenetic data confirm ancestral pattern of the anterior corners of the notum integrated with the separated rhinophores and the oral veil (Figs 1, 3, 7 and 8) for Tritonioidea and Dendronotoidea. This conclusion is further supported by the currently widely accepted close relationship of the nudibranch molluscs with the group Pleurobranchida [2, 25, 122]. All members of Pleurobranchida do not have paired branched dorsolateral appendages, but possess distinct notum (including anterior parts). Therefore, any reductions of the notum including its anterior corners are derived states. Pleurobranchids also have enrolled rhinophores and a lateral gill. The lateral gill is completely reduced in all non-dorid nudibranchs [2, 24]. The genera *Doridoxa* and *Heterodoris* and tritoniid *Tochuina* are lacking the lateral gill, but by patterns of the separate rhinophoral sheaths integrated to the oral veil and presence of the anterior notal corners demonstrate similarity to the enrolled rhinophores of Pleurobranchaeidae integrated into the oral veil [25].

The enigmatic nudibranch *Doridoxa* has always been considered as a crucial taxon to produce a general classification of the nudibranch molluscs [107]. In the present study *Doridoxa* and another poorly known but externally similar taxon *Heterodoris* were included into a broad-scope phylogeny together with the family Tritoniidae. Phylogenetic analysis evidently shows that Tritoniidae evolutionary close to Arminidae, *Doridoxa*, *Heterodoris*, and *Doridomorpha* (Fig 1A). Previous morphological consideration that *Heterodoris* does not belong to the family Arminidae [123] is confirmed in the present study by the molecular data (see also details in the remarks above). This is important because currently *Heterodoris* formally was included into the family Arminidae [124], possibly because it was mentioned in a morphology-based study on the species of *Armina* and *Dermatobranchus* [125].

In a previous analysis [108] *Doridoxa* (without inclusion of *Heterodoris*) was placed with a lower support in a basal position to the majority of the non-dorid nudibranch families. The present analysis places Doridoxidae and Heterodorididae as a sister group to Arminidae (Fig 1A). This is new evidence that the joined rhinophores in Arminidae is a secondary condition, as it was proposed before [25]. The secondary joining of the rhinophores in Arminidae is accompanied by a correlative transversal folding of the oral veil (Fig 1B). This special feature was attested for the majority of arminids [123, 125], but remained morphologically unexplained. The present phylogenetic data support morphological transformation of the ancestral broad oral veil with a smooth (or a finely tuberculated) frontal edge into various number of the distinct elongate processes. To explain this process clearly, a scheme of the potential morphological transformations of the rhinophores and oral veil that supported by the present phylogenetic data is given on the Fig 1B. The oral veil and its derivates are in yellow colour. In a recent study [24] we showed using phylogenetic analysis and ancestral character state reconstruction that pair of oral tentacles can be evolutionary reversed into oral veil-like structure via *paedomorphosis*. In this study we show a potential way of the evolutionary formation of the oral tentacles (as one of the key novelties of the nudibranch molluscs) in course of the process of *peramorphosis* (Fig 1A and 1B) (for a definition of the term see [24]). In support of this implication, within the family Tritoniidae (which usually possesses a broad oral veil), a genus *Marianina* acquired a pair of long, tentacle-like oral processes, whereas other oral veil appendages were considerably reduced (Fig 1B). These phylogenetic data evidently supported the independent formation of the oral tentacles in several major nudibranch lineages. The partly enrolled anterior corners of the oral veils of Pleurobranchida are present in Tritoniidae and probably were additional precursors for the formation of the oral tentacles. Here we also evidently show that another enigmatic family Doridomorphidae (with a single genus *Doridomorpha*) is nested together with *Doridoxa* and *Heterodoris* clades (Fig 1A). Doridomorphidae thus secondarily acquired dorid-like external shape but via a different ontogenetic mechanism: the oral veil of ancestors of *Doridomorpha* was completely merged with the frontal part of the notum (Fig 1B, the derivates of oral veil coloured in yellow in every depicted taxon, including *Doridomorpha*). In true dorids the oral veil is instead enclosed by the frontal notal edge [126]. *Doridomorpha* does not show any distinct oral veil around the mouth under the putative “anterior notal edge” [127, 128]. This is a direct morphological indication that oral veil in Doridomorphidae was fused with the notum. The frontal notal edge of *Doridomorpha* is anterior part of the modified and fused oral veil. In dorids the frontal edge instead is the true notum and separate oral veil is placed under the frontal notum [126].

A division of the traditional dendronotacean nudibranch into superfamilies Dendronotoidea and Tritonioidea is used currently [129]. These superfamilies are highly disbalanced by its taxonomic composition. Dendronotoidea includes eight families (Bornellidae Bergh, 1874, Dendronotidae Allman, 1845, Dotidae Gray, 1853, Hancockiidae MacFarland, 1923, Lomanotidae Bergh, 1890, Phylliroidae Menke, 1830, Scyllaeidae Alder et Hancock, 1855, Tethydidae Rafinesque, 1815). Tritonioidea contains only single family Tritoniidae, supposedly related to Arminidae [4, 117]. The present study is also contributed to this problem. By inclusion the molecular phylogenetic data in analysis on *Doridoxa* and *Heterodoris* was shown that these taxa (without branched dorsolateral appendages) together with Doridomorphidae and Arminidae come as a sister clade to tritoniids (Fig 1A). The tritoniid genus *Tochuina* is similar to the non-tritoniid *Doridoxa* and *Heterodoris* by the rhinophoral and radular patterns but already possesses numerous small dorsolateral branched appendages (secondary gills) at the edge of the notum (Figs 3, 7 and 8). The secondary gills (often represented by dichotomously branched appendages) are a key external character of the family Tritoniidae and a majority of the traditional dendronotacean nudibranchs [2]. According to the present molecular phylogenetic data, the small branched dorsolateral appendages of *Tochuina* could be evolutionary predecessors for the large distinct dorsolateral appendages in other tritoniids (Figs 1 and 8). Antarctic tritoniid *Tritoniella* (without evident branched appendages) is sister to the North Pacific genus *Tochuina* (Fig 1A). It may imply that acquisition of the branched appendages for more efficient respiratory function took place among large-bodied basal tritoniids independently from other traditional dendronotaceans. However it will require special explanation because considerable ontogenetic similarities between radulae of Dendronotidae and Tritoniidae [78, 79] contradict to the paraphyly of traditional dendronotaceans. The unstable phylogenetic position of the tritoniid *Marionia* (with distinct branched appendages) either basal to all tritoniids [19, present study] or rendering Tritoniidae paraphyletic [5, 72, 92] also does not support this scenario. The tracing of the dorsolateral appendages and secondary gills evolution is therefore important both for the family Tritoniidae phylogeny and for a broad-scope understanding of the evolutionary pathways among nudibranch molluscs. In the present study we reconstruct the character evolution for secondary gills in Tritoniidae and Dendronotoidea applying Mesquite software [37] (Fig 8). We conclude that another scenario for the secondary gills evolution is also plausible. This scenario implies that common ancestor of a monophyletic group that includes all traditional dendronotaceans plus Doridoxidae, Heterodorididae, Doridomorphidae and Arminidae already acquired small respiratory dorsolateral appendages (Figs 1 and 8). These ancestral respiratory structures could be represented by not yet completely differentiated appendages at the edge of the notum. Such appendages could combine both small branched processes (as in the modern *Tochuina*) and short cerata-like structures, sometimes partly branched (as in the modern *Lomanotus*) (Fig 8). Further evolutionary differentiation led to appear distinctly branched appendages in Tritoniidae and Dendronotoidea from one hand and elongate cerata in the aeolidacean lineage on the other hand ([24, 73]; present study, Figs 1 and 8; see details of the coding of the character states in the legend to Fig 8). Both branched and non-branched appendages serve as protective structures and secondary gills since they increase an effective body surface for respiration [1, 86, 130, 131]. The appendages can be reduced in various nudibranch lineages when the respiration remains efficient without special structures. An evident case of the reduction of the branched appendages and a secondary formation of the cerata-like structures is occurred in a paedomorphic tritoniid genus *Marianina* (body length up to 15 mm) (Figs 1 and 8). A very small dorid nudibranch *Vayssierea* (a maximal length 6 mm) is entirely lacking any gills [74]. A majority of Dendronotoidea and Tritoniidae (of various sizes) have no distinct notal edge but possess branched appendages (Fig 8). Therefore, distinct notal edges of the gill-less *Doridoxa* and *Heterodoris* serve as a respiratory structure. The oxygen-rich environment of cold waters [132] can promote gigantism [133, 134]. However, among cold-water species of the family Tritoniidae only an exceptionally large-sized *Tochuina* (up to 300 mm in length) possesses small branched appendages and distinct notal edges at the same time (Fig 3H–3L). The model species *Tritonia tetraquetra* and *Tritonia exsulans* from northern Pacific are also very large (up to 300 mm), but lacking a distinct notal edge and have large branched appendages (Figs 2D–2F and 4A–4E). Instead, Antarctic *Tritoniella* (up 80 mm in length) does not possess distinct branched appendages, and hence can respire using only ample notum and short unbranched/weakly branched notal processes. Therefore if in the oxygen-rich cold water environment a reducing of body size is occurred, special respiratory structures can become unnecessary. *Doridoxa* and *Heterodoris* two times smaller than *Tritoniella* (up to 30–40 mm in length), and either completely devoid of any special respiratory structures or possesses only weak appendages (Fig 7D, 7K–7M and 7P–7S). Therefore, in the Doridoxidae-Heterodorididae lineage (Figs 1, 7 and 8) a reduction of the more complex ancestral respiratory appendages could be occurred. The irregular short processes along the notal edge in *Heterodoris* (Fig 7P–7S) may represent remnants of more complex ancestral appendages. The highly modified tropical Arminidae instead acquired lamellae under the notal edge as secondary respiratory structures [86, 123] in the warm water oxygen-deficient environment [132, 135]. Thus, based on the available morphological and phylogenetic evidences several evolutionary scenarios for traditional dendronotaceans (including the family Tritoniidae) are possible to propose. Therefore, currently accepted higher level system of the nudibranch molluscs is an interim one and require further investigations.

## Conclusions

A consolidate phylogeny of the nudibranch family Tritoniidae is presented. Large tritoniid that previously was recorded from the Canadian, Russian and US Pacific coasts and commonly used as model system for neurobiological research comprises at least two species. One species is *Tritonia tetraquetra* (Pallas, 1788) and characterizes externally by commonly orange colour without white lines (Fig 2). Another species is *Tritonia exsulans* Bergh, 1894 and commonly has pink or salmon colour with white lines on the oral veil and between dorsolateral appendages (Fig 4). *Tritonia tetraquetra* inhabits a wider geographic and bathymetric range, at least from Oregon in NE Pacific to Kamchatka and the Kuril Islands in NW Pacific, at the depth about 1 m to 700 m (potentially up to 1000 m). *Tritonia exsulans* instead has a restricted geographic range and so far reliably recorded from California to British Columbia in shallow waters, in a range circa 5–100 m. Described from deeper waters (more than 500 m) off Alaska and California *Tritonia diomedea* Bergh, 1894 shares same morphological characters with molecularly confirmed *T*. *tetraquetra* (Figs 1 and 2) and therefore previously [17, 18] was correctly synonymised with *T*. *tetraquetra*. The complicated history of a long-term confusion between type species of another tritoniid genus, *Tochuina*, *T*. *gigantea* (Fig 3) and real *Tritonia tetraquetra* is cleared. The present phylogenetic analyses for the first time shows that recently described *“Tritonia” nigritigris* from NE Pacific belongs to the genus *Tochuina* and not to *Tritonia* (Fig 1A). Importance of a fine-scale taxonomic diagnostics as the reliable way to integrate complex morphological and molecular phylogenetic patterns in an ontogenetic framework is demonstrated using the case of the family Tritoniidae and presenting the respective taxonomic synopsis. Inclusion of the non-tritoniid taxa *Doridoxa* and *Heterodoris* into phylogenetic analysis (Figs 1, 7 and 8) provides explanation for their similarity to the tritoniid genus *Tochuina* and contributes to the general phylogeny of the nudibranch molluscs.

## Supporting information

S1 Fig Phylogenetic relationships of tritoniidae, doridoxidae, heterodorididae, doridomorphidae, arminidae and dendronotoidea in single COI, 16S, and H3 gene trees inferred by Bayesian inference. Some branches are collapsed.
(TIF)
