# Supplementary figures and images for: Consolidated data on the phylogeny and evolution of the family Tritoniidae (Gastropoda: Nudibranchia) contribute to genera reassessment and clarify the taxonomic status of the neuroscience models Tritonia and Tochuina
Source: PLoS One. 2020 Nov 20;15(11):e0242103. doi: 10.1371/journal.pone.0242103 (PMC7679014; doi:10.1371/journal.pone.0242103)

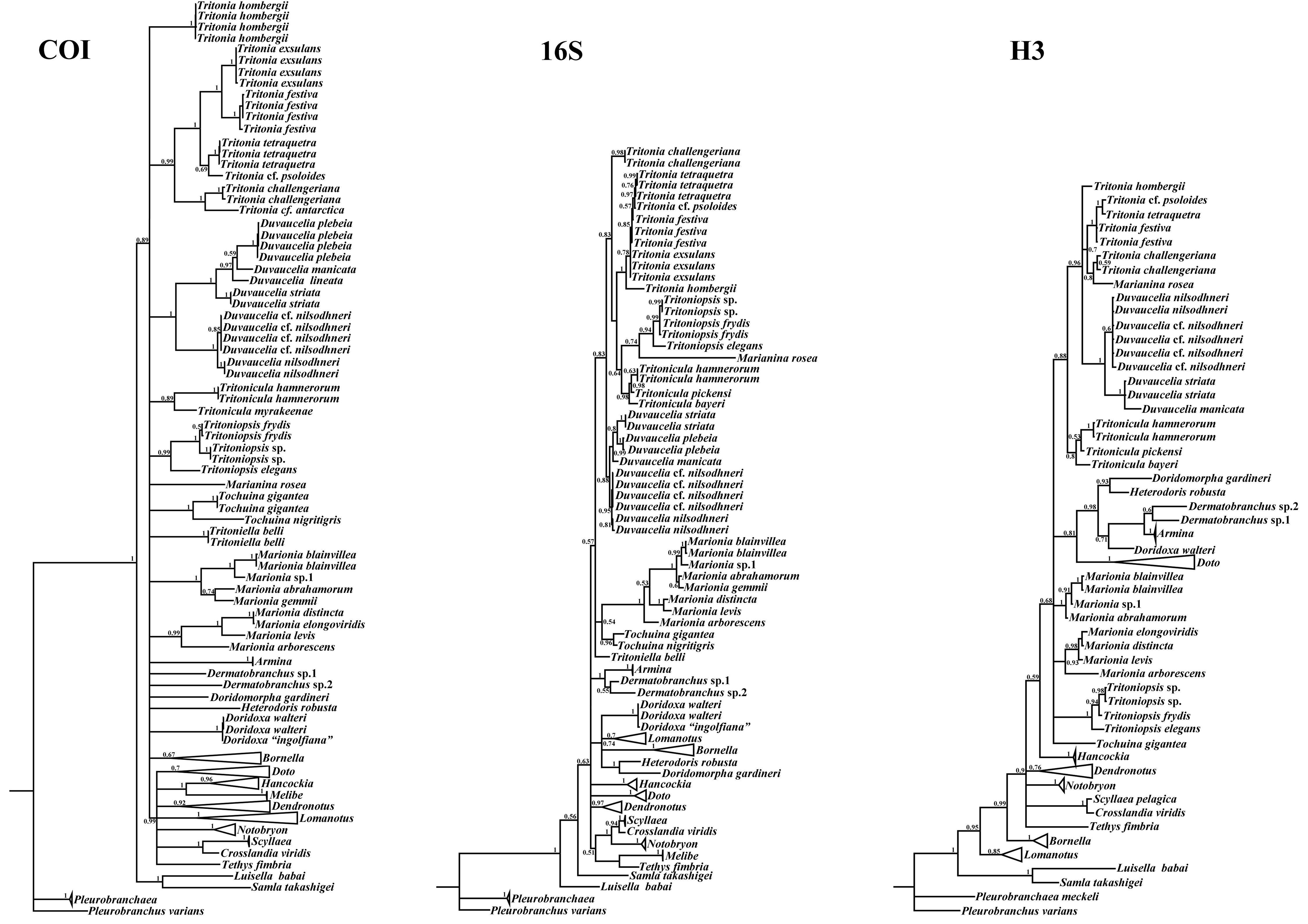

Supplement: S1 Fig — Some branches are collapsed. (TIF) [file pone.0242103.s001.tif]
